# *useeior*: An Open-Source R Package for Building and Using US Environmentally-Extended Input–Output Models

**DOI:** 10.3390/app12094469

**Published:** 2022-04-28

**Authors:** Mo Li, Wesley W. Ingwersen, Ben Young, Jorge Vendries, Catherine Birney

**Affiliations:** 1 General Dynamics Information Technology, Inc., Fairfax, VA 22042, USA; 2 Office of Research and Development, US Environmental Protection Agency, Washington, DC 20460, USA; 3 Eastern Research Group, Lexington, MA 02421, USA

**Keywords:** environmentally-extended input–output, life cycle inventory, life cycle assessment, input–output analysis, environmental impact, open-source software

## Abstract

*useeior* is an open-source R package that builds USEEIO models, a family of environmentally-extended input–output models of US goods and services used for life cycle assessment, environmental footprint estimation, and related applications. USEEIO models have gained a wide user base since their initial release in 2017, but users were often challenged to prepare required input data and undergo a complicated model building approach. To address these challenges, *useeior* was created. In *useeior*, economic and environmental data are conveniently retrievable for immediate use. Users can build models simply from given or user-specified model configuration and optional hybridization specifications. The assembly of economic and environmental data and matrix calculations are automatically performed. Users can export model results to desired formats. *useeior* is a core component of the USEEIO modeling framework. It improves transparency, efficiency, and flexibility in building USEEIO models, and was used to deliver the recent USEEIO model.

## 1. Introduction

Environmentally-extended input–output (EEIO) analysis is a widely used method to identify opportunities for reducing environmental impacts, material use, and waste generation from economic activities or products. EEIO models were developed to calculate direct and indirect environmental impacts in many countries for various applications. In the United States (US), the Environmental Protection Agency (EPA) developed a family of EEIO models, referred to as USEEIO, to support the agency’s Sustainable Materials Management (SMM) program and broader mission [1]. The USEEIO was developed to meet the recommendations of the US source code policy [2], and the recommendations of the National Academies of Sciences, Engineering, and Medicine on reproducibility for computational science [3]. The model was developed with the USEEIO modeling framework [4]. This framework has evolved toward a fuller realization of the recommendations and objectives of transparency, reproducibility, and at the same time, has become more robust.

### 1.1. Background

Prior to the creation of the modeling framework, we reviewed computer languages and modeling tools that would be most pertinent in creating and maintaining USEEIO models. The criteria that the languages/tools should meet included:

- Be free and open-source;
- Use and produce human-readable, non-proprietary data formats;
- Be easily distributed, and installed and used in common computing environments (Windows, MacOS, Linux);
- Support high-level and efficient matrix mathematical operations;
- Be able to be maintained on GitHub or a similar git-based cloud version control platform;
- Have a simple syntax, and support structured programs (modules, sub-route);
- Have an active community;
- Optionally permit graphical user interface development.

Languages and tools that were evaluated and found suitable included Python, Go, R, Julia, Scilab, Java, and Jupyter. Python was initially selected based on it being used by the research team to reassemble the openIO model (See SI2 of [1]), because of its rapidly developing libraries for data science, and its growing usage in the life cycle assessment community. An open source Python package for USEEIO assembly called the input–output model builder (iomb) was the first tool created for the USEEIO modeling framework. The *iomb* required that users independently generate all model economic, environmental, and indicator components in standard .csv data files, with type-specific formats. The first USEEIO model [1] was assembled with the *iomb*. However, collecting various data components and preparing them in correct formats in a way that is reproducible is a challenging process. A simple Python package used to organize some of the USEEIO input data for feeding into the *iomb*, called *useeiopy*, was later developed to assist users with assembling these data to recreate USEEIO models. Nevertheless, the acquisition and transformation of core economic input data were not performed with the package. These data, along with the indicators and environmental data, were each prepared with a large set of independent Excel® models that were not managed in a version control system and were unique to a given USEEIO model. As a result, a lot of core data, such as industry output, lists of environmental flows, and economic data, were replicated across these various Excel® models, which also posed a risk of lack of synchrony or data errors and required more labor to update. Some of the Excel® models of the environmental data grew large enough to consume all available RAM on a typical scientific laptop computer (8–16 GB), which made operation slow and increased the risk of program failure. The R language, which has many similarities in data science applications to Python [5], was also used in USEEIO model component development, initially to retrieve and process larger environmental datasets such as the National Emissions Inventory and USDA chemical use survey. The use of the R language in USEEIO model component preparation continued to grow as USEEIO expanded to cover new datasets, such as the addition of the waste datasets in v1.2 [6], and was used to assemble the simplified two-region USEEIO state models [7]. The early work in the USEEIO modeling framework in R culminated in a set of interdependent R scripts coupled with the *useeiopy* and *iomb* packages that:

- Retrieved and processed Bureau of Economic Analysis (BEA) input–output tables and industry gross output;
- Performed flow and sector mapping using stored .csv files;
- Used a model build script, specific to a given model version, for formatting and writing all the core economic direct requirements and market shares matrices, the environmental and indicator components, and demand vectors for use by *useeiopy/iomb* to assemble the model;
- This work was captured in v0.1 of the USEEIO modeling framework [8].

While the USEEIO modeling framework continued to evolve, a need grew for an increased variety of USEEIO models for various applications. USEEIO was rebranded as a “family of models,” rather than just a single, national US model. An increasing number of contributors and users provided additional evidence of the challenge of synchronizing and fully recreating the latest models. Experience gained in other tools, including in Standardized Emission and Waste Inventories (StEWI) [9] and ElectricityLCI [10], also provided examples of more transparent and reproducible model build paths from data acquisition through to final output, as well as embedded model validation procedures. In response to these needs and challenges, the *useeior* R package was developed, along with a versioning scheme [4] for USEEIO models. *useeior* replaces the *iomb/useeiopy* tools and integrated fully with tools in the USEPA ecosystem of tools for industrial ecology [11].

### 1.2. Overview

*useeior* is an R package for building and using USEEIO models. It was created following the R packages design manual [12] and has advantages including, but not limited to, clear help pages for functions, convenient build checks, explicit dependency installation, and, most importantly, ease of sharing. Consolidating USEEIO model construction in *useeior* not only provided full transparency of data, but also ensured reproducibility of the model.

*useeior* is actively developed and maintained in a public GitHub repository (https://github.com/USEPA/useeior, (accessed on 14 March 2022)), with a primary focus on constructing and enabling technical use of USEEIO models. Using GitHub as the project repository not only allows for a built-in method of version control, but also provides automated build and test checks for *useeior*, utilizing the continuous integration and continuous delivery (CI/CD) platform by GitHub Actions [13]. A configured GitHub Actions workflow, defined by R-CMD-check.yaml in the ’.github/workflows/’ folder, is triggered when an event occurs in the repository, such as a pushed commit or a pull request being opened and updated. In the workflow, a job to execute *R CMD check* will “run in sequential order or in parallel inside its own virtual machine *runner*, or inside a container” [13]. The *R CMD check* examines if the requirements for successfully building the R package are met, such as code, R dependencies, and documentation, and if the validation on a selection of models is successfully completed [12]. This check is a fundamental and easy-to-use quality assurance (QA) tool for *useeior*, and can be combined with more configured workflows to serve as the QA of *useeior*.

*useeior* builds USEEIO models according to a given model configuration/specification and optional hybridization specification, e.g., disaggregation and aggregation, and returns the primary output, *model* object. A limited set of model specifications and associated hybridization specifications for EPA-validated models are included in the ‘format_specs’ folder in the package. The package offers various functions for calculating, validating, visualizing, and writing out models and/or their components.

In *useeior*, underlying input-output (IO) tables, economic gross output data, and chain-type price indices (CPI) compiled by BEA are downloaded and pre-saved in native R data formats (.rda) in the ‘data/’ folder, using the *usethis* package [14], and made available for immediate use; other critical datasets, including sector crosswalk tables that map BEA sectors to the North American Industry Classification System (NAICS) industries, BEA sector code and name correspondence tables, and configuration files for model component attributes are also prepared and saved as .rda files in the ‘data/’ folder. Functions used to download, format, and save the .rda files are available in the ‘data-raw/’ folder. Model aggregation and disaggregation specifications, as well as supporting data, are available for optional use in the ‘inst/extdata/’ folder. Environmental flow data generated by FLOWSA [15], and life cycle impact assessment (LCIA) characterization factors generated by the LCIA formatter [16], can be specified for inclusion in a model and retrieved from the EPA Data Commons. Therefore, to build desired USEEIO models, users do not have to prepare data or LCIA factors; instead, they only need to choose from available models in *useeior* to allow model building functions to construct the desired EEIO models. For advanced users, *useeior* can take user-defined model specifications, and the accompanying data and metadata files, then construct EEIO models using the same model building functions. Detailed instructions are found in the Wiki page in the GitHub repository.

Once a USEEIO model is successfully built, a set of validation steps can be performed to ensure the model is correctly calculated. Then, the model can be conveniently exported to .csv files, Excel® workbook (.xlsx), .bin format, or .json format, which suit various applications. Furthermore, users use built-in visualization functions to inspect and compare the model including matrix coefficients, indicator scores, and sector ranking.

*useeior* was developed and deployed in an iterative pattern, and the release described here is *v1.0.0* [17]. To use *useeior*, it is recommended to install it from GitHub, then load it upon successful installation.

install.packages(“devtools”)

devtools::install_github(“USEPA/useeior@v1.0.0“)

library(useeior)

*useeior v1.0.0* is capable of building USEEIO v2.0 models [18] and variants, such as *USEEIO v2.0.1s.* In the model name, ‘USEEIO’ is the main model name indicating the model is a US (single-region) model; ‘v2.0.1’ is the major (‘v2’) + minor (‘.0.1’) version number; and ‘s’ indicates that the IO data level of detail is summary. Further explanation about USEEIO model naming is available at versioning scheme [4].

USEEIO models prior to v2.0, including USEEIOv1, and its variants v1.1 and v1.2, were not built with *useeior*. Major advances from USEEIOv1 to v2 models included not only the updated economic data (from 2007 to 2012), and environmental data prepared with improved methods, but also novel methodologies about waste sector disaggregation, final demand vectors, and a domestic form of the model. A high-level summary comparison of content differences between the USEEIOv1 and v2 models is available in technical content of USEEIO models [19].

The objective of this paper is to provide a comprehensive introduction of the novel *useeior* package. This paper serves as the primary documentation of *useeior v1.0.0* and enhances transparency of the package and reproducibility of USEEIO models. Users can follow the explicit guidelines described in the paper to build and use v2.0 and v2.1 national USEEIO models with *useeior*. Details of model construction, calculation, validation, and exporting in *useeior* v1.0.0 are described in Section 2. A single-region, summary level (73 commodities and 71 industries) [20] USEEIO model, *USEEIOv2.0.1s,* was used as the example to demonstrate and discuss selected results that can be generated from *useeior* v1.0.0 in Section 3.

## 2. Materials & Methods

The complete model building process in *useeior v1.0.0* requires the following six successful steps and returns a *model* object as the primary output:

1. Initialize model;
2. Load IO data;
3. Load and build satellite tables;
4. Load and build indicators;
5. Load demand vectors;
6. Construct EEIO matrices.

For hybrid models, hybridization processes such as aggregation and/or disaggregation of sectors are incorporated in step 2 and 3.

To simplify the model building process, the six steps are integrated into a wrapper function, *buildModel,* that builds a USEEIO model in one line of code, and provides logging of the build process using the *logging* package [21]:

model <- buildModel(modelname)

Before building a model, it is recommended to check if the model is available in *useeior* using this function:

seeAvailableModels()

*useeior v1.0.0* comes with nine built-in national models (Table 1), and their configuration files are found in the ‘inst/extdata/modelspecs/’ folder.

Model configurations are stored in relevant .yml (interchangeable with .yaml) files. YAML is a simple text-based format used to store configuration data across the USEEIO tool ecosystem [11]. *useeior* uses the *configr* package [22] to parse YAML files.

### 2.1. Model Initialization

The first step in the model building process is to initialize the model according to the input model name, and paths to configuration files if provided. This step establishes the scope, e.g., single-region or two-region, and focus, e.g., specific or all environmental impacts, of the model.

model <- initializeModel(modelname, configpaths)

If the desired model is available in *useeior,* it is initialized based on *modelname* only, i.e., configpaths = NULL. This loads the built-in model configuration .yml file with that model name, and related aggregation/disaggregation configuration, and .yml and data .csv files.

Alternatively, a user-customized model can be initialized as long as its configuration files, including model and related hybridization configuration (in .yml format only), as well as data files (in .csv format only), are prepared following the format of configuration and data files of the available models. All configuration and data files must be accessible in the user directory specified in model configuration, i.e., configpaths = user_directory.

Model initialization returns a model object in list form that contains two elements: model specs and crosswalk. Specs stored the model specifications sourced directly from the model configuration files include:

- Basic information including the name of the model, model region acronym, model type, and pointers to hybridization, such as aggregation and disaggregation;
- Basic IO specifications including base IO schema, base IO level, IO year, model region acronym, IO data source, base price type, base with redefinitions or not, commodity or industry type, and scrap included or not;
- Satellite table specifications including the name of environmental satellite tables, years represented by flows included in the satellite table, path of the source file, source category used for sectors in the satellite table, year and level of resolution of the sectors, original flow source, function name for additional processing of the satellite table, and metadata of satellite table;
- Indicator specifications including name, code, group, and unit of indicator, path of the source file, function name and parameters for additional processing of the indicator, and metadata of indicator; and
- Demand vectors specifications including a pointer to default demand vectors (i.e., a production vector, a consumption vector, and domestic version of the two vectors), and optional demand vectors, such as household purchase that contains name, type, year, system, and location information.

The crosswalk is a sector correspondence table of five columns for sets of BEA and NAICS codes, and any custom codes used in the current model:

1. NAICS—2- to 6-digit NAICS codes (7–10 digit codes exist for manufacturing and mining industries);
2. BEA_Sector—code used at the BEA sector level;
3. BEA_Summary—code used at the BEA summary level
4. BEA_Detail—code used at the BEA detail level;
5. USEEIO—code used at the model level of detail, including any adjustments for hybridization.

The crosswalk is a fundamental table in the *useeior* model building process, as it connects the BEA and NAICS classification systems, which have notably different commodity and industry sectors, and enables mapping from one system to the other. An example of the crosswalk is presented in Table S1 in the Supplementary Information (SI). In *useeior v1.0.0,* the crosswalk is built based on 2012 BEA and NAICS codes, with an inclusion of 2007 NAICS codes, according to the 2012 to 2007 NAICS concordance by Census Bureau [23]. The correspondences between BEA and NAICS codes were adopted from the BEA–NAICS relationship table, published in national IO accounts by BEA [20], which presents a hierarchy of the BEA codes at sector, summary, and detail levels, as well as how each level relates to the NAICS code structure. Two adjustments were applied to the original BEA–NAICS table in order to create a crosswalk that captured all correspondences between BEA and NAICS sectors:

1. For BEA codes not aligned with specific NAICS industries, their correspondences are approximated after careful inspection and comparison of their definitions in the BEA and NAICS systems;
2. For BEA codes that do not have correspondences with the complete hierarchy of NAICS codes (2- to 6-digit), the correspondences are extended to all related NAICS codes, based on the Census 2- to 6-digit NAICS code table [24].

With a complete crosswalk, *useeior* successfully and seamlessly harmonizes economic and environmental data that are categorized by BEA, NAICS, and original classifications.

### 2.2. Economic Input–Output Data

In *useeior*, the most recent IO data are the form of “Make” (showing the production of commodities by industries) and “Use” (showing the consumption of commodities by industries and by final demand) tables compiled by BEA [20], saved in native R data formats, .rda, via automated downloading and writing functions. These tables are available at three levels of sector resolution: “Detail” (405 commodities by 405 industries), “Summary” (73 commodities by 71 industries), and “Sector” (17 commodities by 15 industries).

The summary and sector levels tables are released annually by BEA, while the detailed tables are produced roughly every five years, with 2012 representing the most recent release [20]. Therefore, in *useeior v1.0.0,* the summary and sector make and use tables are available for years 2010–2018, while the detail tables are only available for the year 2012.

The Make and Use tables compiled by BEA were available “before redefinition” and “after redefinition”. Redefinition adjusts secondary products “from the industry that produced it to the industry in which it is primary”. [25] In *useeior*, “before redefinition” tables are used, as they are more aligned with the majority of environmental data that reflect the original industry activities that occurred [1].

Additionally, the use tables are available in producer price and purchaser price. Existing model configuration files in *useeior* used the use tables in producer price. *useeior* provides the option to convert the model from producer to purchaser price, with additions of trade and transportation margins [26].

In EEIO modeling, the direct requirements matrix *A* and domestic direct requirements matrix *A_d_* can be derived from the Make and Use tables to build EEIO models in two forms: industry-by-industry or commodity-by-commodity. *useeior* is capable of building industry (industry-by-industry) and commodity (commodity-by-commodity) models. The former was the most suitable for EEIO models, with a focus on industries and environmental impacts from related producing processes; the latter was most relevant for EEIO models concerned with products and services and their associated materials. A direct requirements matrix derived based on two distinct assumptions, industry–technology and commodity–technology, yields different results. The former assumes that all commodities produced by the same industry have the same input structure, while the latter assumes that each commodity has a unique input structure, regardless of the industry that produced it [25]. *useeior* v1.0.0 implements the industry–technology model.

Other economic data, including multi-year economic gross output, gross output chained price index, the margins table, and the import matrix, are also prepared and made available for immediate use in *useeior*.

Model IO data are loaded upon model initialization and with paths to configuration files if provided.

model <- loadIOData(model, configpaths)

After this step, the *model* object is expanded to include core IO data and related metadata for building the desired model, including:

- The Make table in industry-by-commodity form;
- Use and domestic use tables split into intermediate consumption, final demand, and value added in commodity-by-industry and commodity-by-component forms (note: domestic use table = use table – import matrix);
- Commodity and industry output in model year, as well as in a range of multiple years in commodity-by-year and industry-by-year forms;
- A Margins in commodity-by-margin-sector form disclosing producer price, trade (retail and wholesale) + transportation cost, and purchaser price of each commodity, used for converting from producer price to purchaser price, and vice versa;
- Metadata of the IO data-code, name and group details about commodity, industry, final demand component, value added component, and margin sector;
- IO data and metadata of hybridization if pointers to hybridization, such as aggregation and disaggregation, were not NULL in model specifications.

It is during this step of the model building process that custom hybridization of the model object occurs, when specified in the model configuration file. Currently, *useeior* only supports hybridization in the form of model aggregation and disaggregation, though support for other forms of hybridization is in progress. For model aggregation, the user only needs to input one additional .yml file to specify which sectors are to be aggregated. For model disaggregation, several additional input files need to be provided:

- A .yml file containing a list of sectors, including the sector to be disaggregated and the new sectors that will take its place;
- Two .csv files for the Use and Make tables (one for each) that specify the allocation values from the original to the disaggregated sectors. If the user does not have the data available to provide the allocation values, both .csv files can be omitted and the disaggregation proceeds uniformly based on the number of sectors specified;
- A .csv file providing inputs for disaggregation of the satellite tables (see next section).

For all aggregation and disaggregation procedures, the commodity and industry sums are compared for equality across the Use and Make tables to ensure that the economic balance is maintained. If user inputs result in an unbalanced model, *useeior* attempts to balance the tables using a RAS approach. If this balancing is unsuccessful, the program execution halts and requests revised input files that will result in a balanced model.

### 2.3. Environmental Data and Satellite Tables

*useeior* characterizes the amount of environmental releases/losses, resource use, waste generation, and employment by model-specified industry, through the use of national totals of flows by NAICS industry and the crosswalk created in Section 2.1. National totals of flows by NAICS industry data used in *useeior* can be generated by a Python-based tool called FLOWSA [15], which structures data in a flow-by-sector (FBS) format. The latest version of FLOWSA, v1.0.1, delivers FBS data that covers a variety of flow types, including criteria and hazardous air emissions, point source industrial releases to water and soil, use of land, use of water, and employment. To support impact assessment and a consistent flow naming system across data sources, flows in FLOWSA conform to the Federal Elementary Flow List (FEDEFL) [27]. The FBS data to build the models specified in *useeior* v1.0.0 are retrieved from the EPA Data Commons [28] via automated functions in *useeior*.

These flow-by-NAICS-industry data are transformed into flow-by-model-sector format, and loaded as satellite tables via the NAICS-to-BEA crosswalk. Value added by BEA industry are also considered flow data, but are directly loaded from the IO data that was added in the previous step.

model <- loadandbuildSatelliteTables(model)

A Satellite Tables component is added in the *model* object after this step. In the new component, the satellite tables are stored in *totals_by_sector*, while flow metadata were stored in *flows*. Each type of flow had a designated satellite table. Following the loading of all satellite tables, *useeior* identified instances of flows reported by the same sector from multiple satellite tables as a means to avoid double counting. All satellite tables were formatted into a standard structure that included the following columns:

- Flow name, context, universally unique identifier (UUID), amount, unit, location, and data year;
- Sector code and name;
- Data quality scores for data reliability, temporal correlation, geographical correlation, technological correlation, and data collection.

Flow metadata displays unique flows found across all satellite tables with names, contexts, UUIDs, and units were sourced from FEDEFL.

Satellite, or *totals_by_sector*, tables provide a full picture of flows from and to the environment. They are used to calculate impact coefficients and validate the model.

If aggregation or disaggregation is specified during model build, each satellite table is aggregated or disaggregated upon loading. Specifications for disaggregating environmental data can be provided in one of two ways. If flow totals are provided for a given flow for any of the new sectors, *useeior* replaces the existing satellite table data for that flow with the data in the supplementary disaggregation file. Alternatively, flow ratios can be provided for one or more flows in a satellite table. If the FlowRatio field was supplied, *useeior* disaggregates each flow to the new sectors according to the supplied ratios. In either case, if data for a specific flow are not provided, *useeior* disaggregates the existing satellite table data for that flow proportional to gross industry output of the new sectors.

### 2.4. Indicators and Life Cycle Impact Assessment Characterization Factors

Model indicators quantitatively aggregate the environmental flow data to their corresponding impact categories, through the use of life cycle impact assessment (LCIA) characterization factors. For example, flows of greenhouse gases are valued as carbon dioxide equivalencies. To support the use of environmental flow data retrieved from FLOWSA, standard LCIA characterization factors generated by the LCIA formatter were used to populate model indicators [16]. Users can choose from a number of available methods in the LCIA formatter including the Tool for Reduction and Assessment of Chemicals and Other Impacts (TRACI) [29], ReCiPe [30], etc., to generate LCIA factors that suit their needs. For any model, the method parameter is customizable and can be modified under the indicators section in model specifications (see Supplementary Information S2 for example of model specifications).

model <- loadandbuildIndicators(model)

A new component *Indicators* is added in the *model* object after this step. In the new component, *factors* table presents the LCIA characterization factors linking one unit of the flow to its indicator, while *meta* table includes metadata for the indicators included in the model.

### 2.5. Final Demand

The final demand vectors represent purchases of goods and services by final consumers, including households, investors, and governments. This function generates final demand vectors specified by model specs:

model <- loadDemandVectors(model)

A new component *DemandVectors* is added in the *model* object after this step. In the new component, *vectors* contain numeric vectors of final demand, while *meta* table includes metadata for the demand vectors included in the model.

In *useeior,* two primary final demand vectors, a production vector and a consumption vector, plus the domestic version of the two vectors are prepared as default vectors for all models. They are the same final demand vectors described in the USEEIO v2.0 documentation [18]. Additional demand vectors, such as household purchases, can be added in *DemandVectors* if declared in model configuration.

### 2.6. EEIO Matrices Construction

The last step to build a complete USEEIO model is to construct EEIO matrices based on previously loaded IO, satellite, and indicator tables.

model <- constructEEIOMatrices(model)

Satellite tables are first combined into one *totals_by_sector* table, *TbS*. The *TbS* table is then used to calculate coefficients-by-sector and generate a *CbS* table.

IO tables loaded by previous step are formed into matrices and vectors with standard notations:

- The Make matrix, *V*, is an industry x commodity matrix with amounts in commodities in year USD produced by industries;
- The Use matrix, *U*, is a commodity x industry matrix with total amounts in model year USD of commodities used by industries for intermediate production, or used by final consumers. *U* also includes commodity imports, exports, and change in inventories as components of final demand, and value added components as inputs to industries;
- The domestic Use matrix, *U_d_*, is a commodity x industry matrix that provides commodity and value added use totals by industries, and final demand, only from the US;
- The commodity output vector, *q*, and the industry output vector, *x*, contain economic gross output in model year US dollars;
- The market shares matrix, *V_n_*, is a *q* normalized form of *V*, also in industry x commodity format;
- The commodity mix matrix, *C*_*m*_, is an *x* normalized and transposed form of *V* in commodity x industry format.

Model matrices are then prepared. The direct requirements matrix, *A*, is a sector x sector matrix that contains in each column, *i*, the direct sector inputs required to produce USD 1 of output from sector *i*. *A* is created from the normalized forms of the model make, *V*, and use, *U*, tables in one of two ways, depending on if the model type was set to be commodity or industry (Equations (1) and (2)).

(1) $$A(c)=U_{n}*V_{n}$$

(2) $$A(i)=V_{n}*U_{n}$$

(3) $$U_{n}=U{\hat{x}}^{-1}$$

(4) $$V_{n}=V{\hat{q}}^{-1}$$

The domestic direct requirements matrix, *A_d_*, is a sector x sector matrix that provides direct sector inputs per dollar sector output, only from the US. Similar to *A*, *A_d_* is created from the normalized forms of the model Make, *V*, and Use, *U_d_*, tables in one of two ways, depending on if the model type is set to be commodity or industry in the model configuration file (Equations (5) and (6)).

(5) $$A_{d}(c)=U_{d_{n}}*V_{n}$$

(6) $$A_{d}(i)=V_{n}*U_{d_{n}}$$

(7) $$U_{d_{n}}=U_{d}{\hat{x}}^{-1}$$

The total requirements matrix, *L* (the Leontief inverse of *A*), is a sector x sector matrix that contains in each column, *i*, the total requirements of the respective sectors inputs per 1 USD of output from sector *i*. *L* is obtained from *A*, using Equation (8).

(8) $$L={\left(I-A\right)}^{-1}$$

The domestic total requirements matrix *L_d_* (the Leontief inverse of *A_d_*), is a sector x sector matrix that provides total sector inputs per dollar sector output, only from the US. *L_d_* is obtained from *A_d_*, using Equation (9).

(9) $$L_{d}={\left(I-A_{d}\right)}^{-1}$$

The direct emission and resource use matrix, *B*, is a flow x sector matrix that contains in each column, *i*, the amount of a flow given in the reference units of the respective flow (e.g., kg) per USD 1 output from sector *i*. To obtain *B*, *B*(*i*) is first derived from *E*, a emission x industry matrix of national totals of each flow by industry sector in year *y*, and *x_z,y_*, a vector of gross output by industry in year *z*, given in year *y* dollars (Equation (10)).

(10) $$B{\left(i\right)}_{y}=E_{z}{\hat{x}}_{z,y}^{-1}$$

The industries in the *E* columns match the industries in *x*.

For *x* to be in year *y* USD, the year of the IO data, *x*, must first be price adjusted using Equation (11), where *x_z_* is the year industry output for industry, *i*, in the currency year, *z*, corresponding to the year of the national flow totals.

(11) $$x_{y}=x_{z}*\rho_{z->y}$$

If model type is *Industry*, *B*(*i*) is essentially *B* flow x industry form.

If model type is *Commodity*, *B*(*i*) is transformed with the market shares matrix *V_n_* and becomes *B*(*c*) in flow x commodity form (Equation (12)).

(12) $$B(c)=B(i)V_{n}$$

The original relation between the environmental data in the form of national totals by industry, *E*, and the model economic data uses the model industry output, as described in Equation (10).

The characterization factor matrix, *C*, is an indicator *x* flow matrix that contains in each column, *k*, the characterization factors of the indicators related to one reference unit of flow *k*. The factors in *C* are inherited from indicators, and used to convert and aggregate individual environmental flows, e.g., carbon dioxide, methane, etc., to total impact of the corresponding indicator, e.g., greenhouse gas. The price year conversion matrix, *Rho*, is a sector x year matrix that contains in each column *y* model-IO-year-to-year USD ratios. The price type conversion matrix, *Phi*, is a sector x year matrix that contains in each column, *y*, producer to purchaser price ratios.

Lastly, the following core EEIO matrices are constructed to complete the model.

The direct impact coefficient matrix, *D*, is an indicator x sector matrix that contains in each column, *i*, the direct impact (e.g., kg CO_2_ eq) per USD output from sector *i*. *D* is derived from the multiplication of *C* and *B* in one of two ways, depending on if the model type is set to be commodity or industry (Equations (13) and (14)).

(13) D(c)=CB(c)

(14) D(i)=CB(i)

The direct and indirect flow coefficient matrix, *M*, is a flow x sector matrix that contains in each column, *i*, the direct and indirect amount of a flow given in the reference units of the respective flow (e.g., kg) per USD 1 output from sector *i*. *M* is derived from the multiplication of *B* and *L* in one of two ways, depending on if the model type is set to be commodity or industry (Equations (15) and (16)).

(15) M(c)=B(c)L

(16) M(i)=B(i)L

The domestic form of *M*, *M_d_*, is a flow x sector matrix that contains in each column, *i*, the direct and indirect amount of a flow given in the reference units of the respective flow per USD 1 sector output, only from the US. Similar to *M*, *M_d_* is derived in one of two ways, depending on if the model type is set to be commodity or industry (Equations (17) and (18)).

(17) $$M_{d}(c)=B(c)L_{d}$$

(18) $$M_{d}(i)=B(i)L_{d}$$

The direct and indirect impact coefficient matrix, *N*, is an indicator x sector matrix that contains in each column, *i*, the direct and indirect impacts (e.g., kg CO_2_ eq) per USD output from sector *i*. *N* is derived from the multiplication of *D* and *L* in one of two ways, depending on if the model type is set to be commodity or industry (Equations (19) and (20)).

(19) N(c)=D(c)L

(20) N(i)=D(i)L

The domestic form of *N*, *N_d_*, is an indicator x sector matrix that contains in each column, *i*, the direct and indirect impacts per USD sector output, only from the US. Similar to *N*, *N_d_* is derived in one of two ways, depending on if the model type is set to be commodity or industry (Equations (21) and (22)).

(21) $$N_{d}(c)=D(c)L_{d}$$

(22) $$N_{d}(i)=D(i)L_{d}$$

At this point, a complete USEEIO model is successfully constructed. The environmental impact coefficient matrices, i.e., *B*, *D*, *M*, and *N*, are directly usable for life cycle assessment, input–output modeling, footprint, and related applications.

### 2.7. Matrix Price Adjustment

A coefficient matrix (*B*, *D*, *M*, or *N*) can be further adjusted to desired currency year (e.g., 2018) and price type (e.g., purchaser price) via

matrix_adj<- adjustResultMatrixPrice(matrix_name, currency_year = 2018, purchaser_ price = TRUE, model)

The returned matrix has the same dimensions and format as the original coefficient matrix. *useeior v1.0.0* supports currency year adjustment from 2007 to 2018, to control for the influence of inflation on the model. The conversion from producer to purchaser price is most useful from a consumer perspective, as the purchaser price, i.e., the price paid by consumers, equals to producer prices plus any associated margin, which generally includes distribution, wholesale and retail costs, and price type adjustment from producer to purchaser price.

### 2.8. Model Calculation

Model matrices can be used to calculate life cycle inventory (LCI) and life cycle impact assessment (LCIA) results given a user-specified perspective, demand vector (from *DemandVectors* in the model object or a user-provided vector), and a selected requirements matrix (complete or domestic).

result <- calculateEEIOModel(model, perspective = “DIRECT”, demand = “Production”, use_domestic_requirements = FALSE)

The return result list contains two matrices: either *LCI_d_* and *LCIA_d_,* where *d* indicates the “DIRECT” perspective, or *LCI_f_* and *LCIA_f_*, where *f* indicates the “FINAL” perspective.

The direct perspective calculation associates the total impact with the sectors that produce the given flows (e.g., direct emissions, waste generation, or resource use), while the final perspective calculation associates the total impacts with the final consumption sectors that drives that impact.

The direct perspective LCI, i.e., the *direct* flows matrix, is calculated with Equation (23).

(23) $$LCI_{d}=B\hat{s}$$

where *s*, a scaling vector, is the product of *L* and the given final demand vector, *y*, as shown in Equation (24).

(24) s=Ly

A similar approach is used to calculate the *direct* impacts with the direct perspective Equation (25).

(25) $$LCIA_{d}=D\hat{s}$$

The *direct* + *indirect* flows matrix with the final perspective, *LCI_f_* is calculated with Equation (26).

(26) $$LCI_{f}=M\hat{y}$$

The *direct* + *indirect* impacts are calculated as in Equation (26), but use *U* in place of *M*, as shown in Equation (27).

(27) $$LCIA_{f}=N\hat{y}$$

To calculate any domestic result, the *L_d_* and a demand vector derived from *y_d_* are used. The difference between any full result calculation and the domestic calculation can be used to derive rest of world region results, as in Equation (28).

(28) $$LCIA_{d,RoW}=LCIA-LCIA_{d,US}$$

where *LCIA*_*d,RoW*_ is the contribution from rest of world, and *LCIA*_*d,US*_ is the contribution from the US.

To calculate a flow’s contribution to total impacts of an indicator in a sector, divide the product of the flow’s total impact, in *M* or *M_d,* and its indicator factor, in *C*, by the sum of total impacts in the sector.

flow_impact <- calculateFlowContributiontoImpact(model, sector, indicator, domestic = FALSE)

To calculate a sector’s contribution to total impacts of an indicator, divide the product of the sector’s total requirements, in *L* or *L_d*, and the indicator’s direct impact by flow, in *D*, by the sum of total impacts of the indicator.

sector_impact <- calculateSectorContributiontoImpact(model, sector, indicator, domestic = FALSE)

To calculate the total impact of an indicator passed from one sector to another through purchase, the diagonalized form of the indicator’s direct impact, *D*, is multiplied by the product of total requirements, *L*, and the diagonalized form of the demand vector, *y*. *y* can be a calculated demand vector in *model* or a user-specified vector that has the same dimension with any model demand vector. *sector2sector_impact* is a matrix of total impacts in the form of sector purchased x sector sourced, where negative values are interpreted as reduced impacts.

sector2sector_impact <- calculateSectorPurchasedbySectorSourcedImpact(y, model, indicator)

Margin impacts are calculated via multiplying the normalized impact of margin sector (i.e., retail, wholesale, and transportation sectors) on each commodity by the total impact coefficients (amount per dollar in producer price) of each commodity. As a result, margin impacts are delivered in by-flow and by-indicator forms based on the model *M* and *N* matrices.

margin_impact <- calculateMarginSectorImpacts(model)

Any sector x flow matrix can be normalized by the total of respective flow (column sum) for usage in further applications.

matrix_n <- normalizeResultMatrixByTotalImpacts(m)

Any sector x flow matrix can be aggregated by row or by both row and column to a user-defined sector’s level of detail.

matrix_aggbyrow <- aggregateResultMatrixbyRow(matrix, to_level, crosswalk) matrix_agg <- aggregateResultMatrix(matrix, to_level, crosswalk)

### 2.9. Model Validation

A series of validation functions are available to validate that model calculation results were equivalent to known IO and EEIO identities. As model calculation results would not be expected to match exactly, due to rounding in original datasets, the margin of error is customizable to meet different restrictions, with a default setting of 1% error. Complete model validation checks were performed in ValidateModel.Rmd in the ‘inst/doc/’ folder. Knit ValidateModel_render.Rmd in the same folder to run all validation checks on selected models specified under the YAML header. This returns an .html and a .md file in the ‘inst/doc/output/’ folder containing validation results for each model.

A full model validation is performed via verifying that national flow totals by sector used as inputs to the model can be recalculated using appropriate model components.

model_val <- compareEandLCIResult(model, use_domestic = TRUE, tolerance = 0.01)

This validation was performed using Equation (29).

(29) $$E=B\dot{\chi }L\hat{y}$$

If model is a commodity model, *E* on the left side becomes *E*(*c*), which is the original flow by industry totals, *E*(*i*), put into a flow x commodity form. *E*(*i*), a national total of flow by industry per year, consisting of the concatenation of all the satellite tables described above, and is available for various years. *E*(*c*) is obtained from *E*(*i*) by multiplying its transpose by the commodity mix matrix, *C*_*m*_, and transposing the result (Equation (30)).

(30) $$E(c)={\left(C_{m}E{\left(i\right)}^{\prime }\right)}^{\prime }$$

(31) $$C_{m}=V^{\prime }{\hat{x}}^{-1}$$

*C_m_* is obtained from Equation (31), where *V′* is the transposed model make table, which is normalized by multiplying it by the diagonalized form of the inverse of model output, *x*.

Given the commodity model, the right side of Equation (29) is a slightly modified form of the matrix, calculated using the direct perspective, where *B* becomes *B*(*i*) representing the satellite matrix in industry form from Equation (10).

As the original flow totals in *E*(*i*) may be in varying years, while the model IO data are all in the IO year (e.g. 2012 for USEEIO v2.0), to validate the model, *B*(*i*) requires an output adjustment via multiplication with *χ*, an output adjustment matrix. *χ* is composed of *x*_*s*_ : *x* output ratios and in the same form, as well as rows and column identifiers, as *B*(*c*). The element-wise product of *B*(*i*) and *χ* adjusts *B*(*i*) for the flow year differences, and effectively converts *B*(*i*) into a harmonized IO year form. Before being multiplied with the commodity-based $L\hat{y}$ product, *B*(*i*) is further transformed using Equation (12) into commodity form, *B*(*c*), via the market shares matrix, *V_n_*, obtained from Equation (4).

If *model* is an industry model, *E* on the left side of Equation (29) becomes *E*(*i*), which is the original flow by industry totals, while *B* on the right side becomes *B*(*i*). As *L* and $\hat{y}$ are also in industry form, they can directly times *B*(*i*) and *χ*, then the product is comparable against *E*(*i*) on the left side of Equation (29).

Additional validations were performed to:

- Check that economic output, industry output (if *model* is an industry model), or commodity output (if model is a commodity model) can be recalculated by final demand multiplying the Leontief matrix in Equation (32) or Equation (33).
  econ_val <- compareOutputandLeontiefXDemand(model, tolerance = 0.01)
(32) $$x=L\hat{y}$$
(33) $$q=L\hat{y}$$
- Check that a commodity model’s final demand and commodity output can be recalculated by summing domestic use.
  q_val <- compareCommodityOutputandDomesticUseplusProductionDemand(model, tolerance = 0.01)
(34) $$q=y_{\mathrm{domestic},\mathrm{production}}$$
- Check that total commodity output can be recalculated by industry output via transformation of CPI ratios.

q_x_val <- compareCommodityOutputXMarketShareandIndustryOutputwithCPI-Transformation(model, tolerance = 0.01)

(35) $$q\rho_{c,z->y}=C_{m}x\rho_{i,z->y}$$

Output from the validation functions included the compared objects, their relative differences, passing records, and failing records. Quickly showing whether there are failures, and which sectors failed, is a primary goal of model validation, and provides clear directions to address the failures.

print(paste(“Number of sectors failing:”, model_val$N_Fail))

print(paste(“Sectors failing:”, paste(model_val$Failure$rownames, collapse = “, “)))

### 2.10. Model Exporting

Models can be conveniently exported to .csv files, Excel® workbook (.xlsx), or .bin format, in a user-specified directory that suits various applications. Recently exported model files overwrite existing files by default.

One outlet for the USEEIO model is the USEEIO API [31], which was designed for dynamic access by applications or other uses. This wrapper function exports model components required by the API to a user-specified directory basedir and sub folders.

writeModelforAPI(model, basedir)

To comply with format requirements of the API, model matrices, including *V*, *U*, *U_d_*, *A*, *A_d_*, *B*, *C*, *D*, *L*, *L_d_*, *M*, *M_d_*, *N*, *N_d_*, *Rho*, and *Phi*, are written to .bin files in the ‘basedir/build/data/modelname/’ folder, where *modelname* is the name of the given model. Model demand vectors are written to .json files in ‘basedir/build/data/modelname/demands/’ folder. Model description and metadata of indicators, demands, sectors, flows, and years are written to .csv files in the ‘basedir/build/data/’ folder.

A Python script (https://github.com/USEPA/USEEIO/blob/master/olca/u2o.py, (accessed on 14 March 2022)) is available to generate a fully-linked JSON-LD model compatible with the openLCA JSON-LD schema [32], which leverages the output of writeModelforAPI.

The matrices that are written to .bin files for the API use can also be exported to individual .csv files, by specifying to_format = “csv”. The files can be saved to any user-specified folder, outputfolder, and do not have to be the same basedir in writeModelforAPI.

writeModelMatrices(model, to_format = “csv”, outputfolder)

A consolidated Excel® workbook (.xlsx) may be created to store the model matrices mentioned above, model commodity and industry output (*q* and *x*), model demand vectors, and model sector crosswalk, plus the metadata of demands, flows, indicators, commodities, or industries (depending on if the model was a commodity or industry model), final demand, and value added..

writeModeltoXLSX(model, outputfolder)

A 16-digit hash of the full model object can be created to assign the model object a unique id.

generateModelIdentifier(model)

### 2.11. Model Visualization

Good visualizations present critical analysis and findings in the most effective ways. *useeior* provides a series of visualization functions to showcase fundamental results from the model, and assist further analysis.

Model coefficient matrices such as *N* can be visualized to show coefficients for a given model, or compare coefficients across models. Users choose to view a single indicator (coefficient_name) or multiple indicators at once. They can also remove sectors (sector_to_remove) if a close-up examination on certain sectors is desired.

plotMatrixCoefficient(model_list = list(modelA, modelB), matrix_name = “N”, coefficient_name = “Greenhouse Gases”, sector_to_remove = ““,y_title = “Greenhouse Gases”, y_label = “Name”)

Indicator scores calculated from totals_by_sector and displayed by BEA sector level can be visualized to show scores for a given model, or scores can be compared across models. Users specify the sectors (sector) that interest them in terms of their scores for a given indicator (indicator_name).

barplotIndicatorScoresbySector(model_list = list(modelA, modelB), totals_by_sector_ name = “GHG”, indicator_name = “Greenhouse Gases”, sector = FALSE, y_title = “Greenhouse Gases”)

Model LCI and LCIA results can be visualized to show flows or impacts split by a region, and the rest of the region. For example, users calculate and then visualize impacts associated with domestic consumption as a portion of total consumption in the US.

fullcons <- calculateEEIOModel(model, perspective = “DIRECT”, demand = “Consumption”) domcons <- calculateEEIOModel(model, perspective = “DIRECT”, demand = “Consumption”, use_domestic_requirements = TRUE) barplotFloworImpactFractionbyRegion(R1_ calc_result = domcons$LCIA_d, Total_calc_result = fullcons$LCIA_d, x_title = “Domestic Proportion of Consumption Impact in the US”)

Model LCI and LCIA results can also be visualized to show sector rankings according to given indicators.

result <- calculateEEIOModel(model, perspective = “DIRECT”, demand = “Production”, use_domestic_requirements = FALSE) heatmapSectorRanking(model, matrix = result$LCIA_d, indicators = c(“ACID”, “GHG”, “WATR”), sector_to_remove = ””,N_sector = 20)

Flow data coverage can be visualized to show the presence or absence of flows from the various environmental and employment flow datasets.

heatmapSatelliteTableCoverage(model, form = model$specs$CommodityorIndustryType)

### 2.12. Model Comparison

Comparison between two models was accomplished by executing compare functions in a built-in CompareModel.Rmd in the ‘inst/doc/’CompareModels.Rmd)’ folder. To perform comparison on selected models, use CompareModel_render.Rmd in the same folder to specify model names under the YAML header, then knit the document. This returns an .html and a .md file in the ‘inst/doc/output/’ folder containing comparison results for each model. Currently, only flow totals between two models are compared with built-in function. More comparisons will be added in the future.

model_comparison <- compareFlowTotals(modelA, modelB)

## 3. Results

A single-region, summary level (73 commodities and 71 industries) USEEIO model, *USEEIOv2.0.1s,* is used as the example to demonstrate and discuss selected results in this section. The model is built with 2018 Summary IO data, 2010–2017 environmental flow data and a collection of indicators used with other v2 models like USEEIO v2.0.1–411 [18].

**Figure 1.**
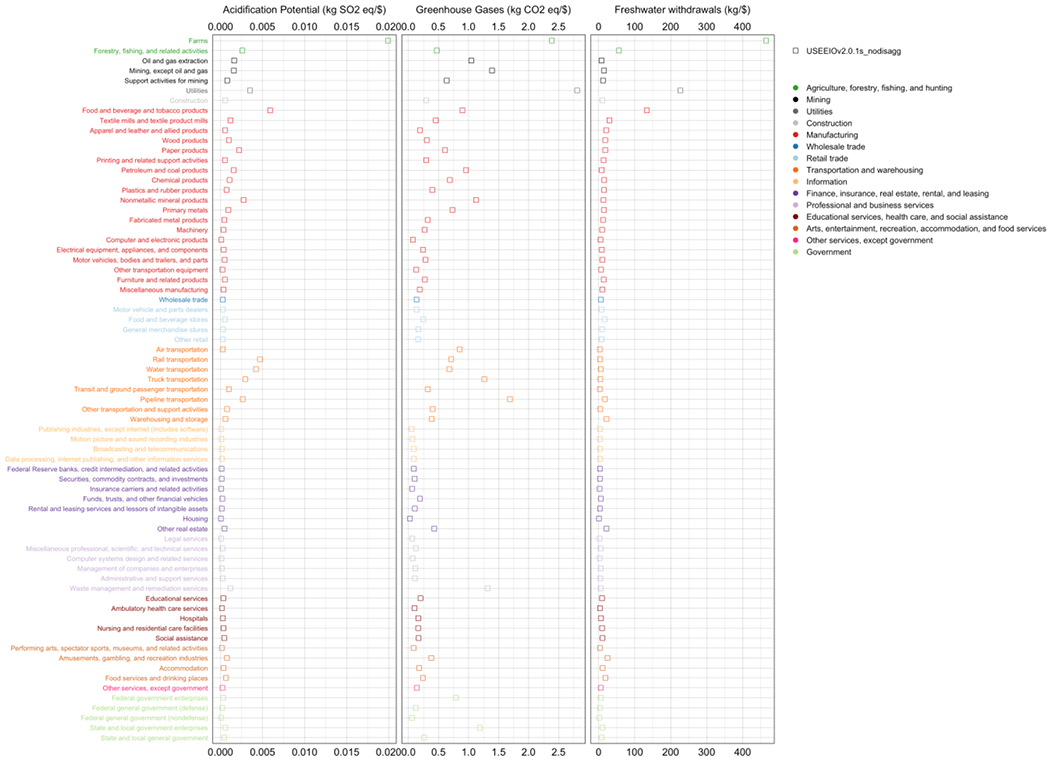
Total impact coefficients by commodity for acidification potential, greenhouse gases, and freshwater withdrawals.

Total impact (direct + indirect) coefficients by sector, i.e., *N* (see Equation (19)), are examined through the *plotMatrixCoefficient* function. Results of three impact categories, including acidification potential (ACID), greenhouse gases (GHG), and freshwater withdrawals (WATR), are presented in Figure 1. It should be noted that coefficients of these impact categories are generated using LCIA characterization factors from the TRACI2.1 method [33]. The *farms* sector has the largest ACID (0.02 kg SO_2_ eq/USD) and WATR (460 kg/USD) coefficient, and the second largest GHG coefficient (2.4 kg CO2 eq/USD)—only smaller than that of the *utilities* sector (2.8 kg SO_2_ eq/USD), which is carbon intensive due to primarily fossil fuel-based electric power generation in the US. *Utilities* also has a notably large WATR coefficient (230 kg/USD), for the same reason. Energy intensive sectors including resource exploitation (i.e., *oil and gas extraction* and *mining*), manufacturing, transportation, and waste management sectors have relatively large GHG coefficients. This illustration provides a clear view of total impact coefficients by sector in the model year. With more environmental flow data over years, multiple snapshots of the illustration could reflect changes in impact coefficients potentially caused by technological advancement in industries, or structural changes in the economy.

Ranking sectors based a composite score of selected total impacts associated with total US demand is an effective means to identify prioritization opportunity in practices, such as the EPA’s Sustainable Materials Management program. Comparing rankings is another form of model validation that incorporates the demand vectors and the indicators, as well as the matrices. The composite score for the rankings is calculated as a sum of fractions of sector impact relative to total impact across all sectors, by each selected indicator. This is represented using Equation (36), where *s* represents this score and *t*, calculated in Equation (37), is a vector of the column sums of the given *LCIA* (see Equation (25)) matrix.

(36) $$s=\left(LCIA{\hat{t}}^{-1}\right)i$$

(37) $$t=i^{\prime }LCIA$$

**Figure 2.**
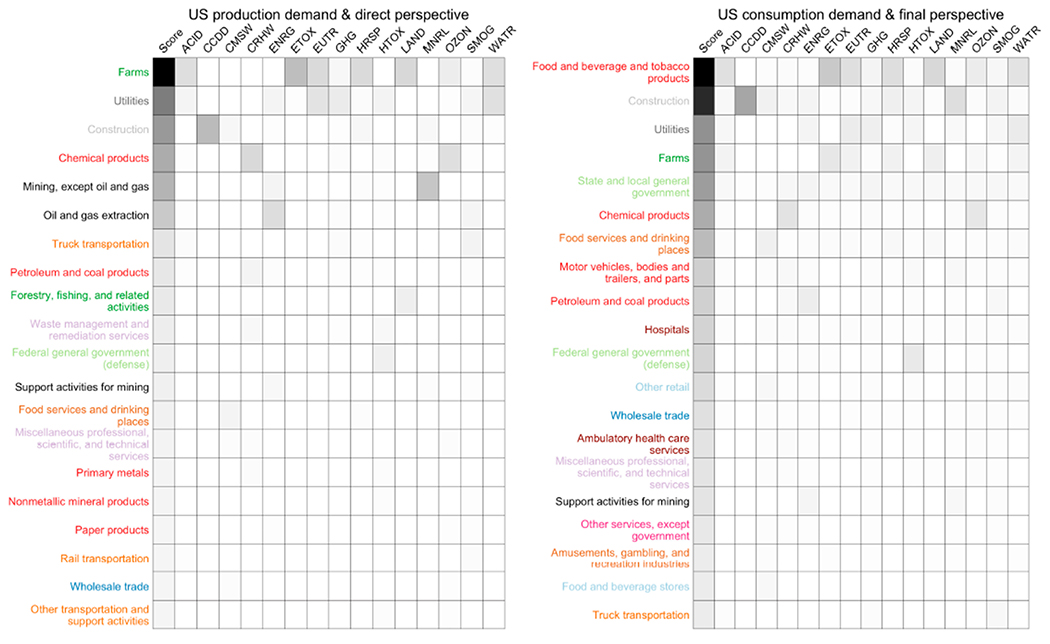
Top 20 commodities by composite impact score for USEEIOv2.0.1s calculated using the total US production demand vector and the direct perspective (**left**), and using the total US consumption demand vector and the final perspective (**right**). Darker shade indicates a relatively higher score. Color of text on the vertical axis follows the color grouping in Figure 1.

The first ranking uses *LCIA_d_* with the US production vector (left in Figure 2), while the second ranking is performed with *LCIA_f_* (see Equation (27)), along with the US consumption vector (right in Figure 2). The sets of commodities in the top 20 from the two rankings (left and right in Figure 2) are nearly identical, with some notable substitutions and some exchanging of places. *Farms, utilities*, and *construction* are in the top places in both rankings, but the orders and the distance (darkness of shade) separating *Construction* from the other commodities are different. *Food and beverage and tobacco products* is not in top 20 in the impact ranking created with *LCIA*_*d*_ and the US production vector (left), but is in the top place in the other impact ranking created with *LCIA_f_* and the US consumption vector, as the latter calculation captures impacts, e.g., human health—respiratory effects (HRSP), associated with the use phase of commodities, e.g., tobacco.

Contribution from the top five flows to total acidification potential in the *Utilities* sector is shown in Table 2. As fossil fuels are still significant resources used by the *Utilities* sector in the US, and sulfur dioxide and nitrogen dioxide emissions are the main cause for acidification potential, it is natural that they are the top two flows, and together contribute to more than 96% of the total impact. The other contributing flows are ammonia, sulfuric acid, and hydrofluoric acid, which together contribute to less than 4% to the total impact.

Contribution from the top five sectors to direct freshwater withdrawals in the *Food and beverage and tobacco products* sector is shown in Table 3. The *Farms* sector contributes the most, because most inputs to the *Food and beverage and tobacco products* sector come from *Farms*. The *Utilities* sector is in the second place, with less than 5% contribution, most likely due to water use in processing food, beverages, and tobacco products. The other contributing sectors are the *Food and beverage and tobacco products* sector itself, which relates to by-products in the sector, the *Forestry, fishing, and related activities* sector, which relates to seafood produced in the food sector, and the *Fabricated metal products* sector, which most likely relates to canning of food and beverage.

## 4. Conclusions

The USEEIO modeling framework requires model building tools to be free, open-source, and easily distributed, installed, and used in common computing environments. The programming languages behind the tools should have simple syntax and an active user community; support structured programs, and high-level, efficient matrix mathematical operations; use and produce human-readable, non-proprietary data formats; and be able to be maintained on GitHub, or a similar git-based cloud version control platform. Among suitable languages, Python was initially selected to create two packages, *iomb* and *useeiopy*, for USEEIO assembly and organizing some of the USEEIO input data, respectively, but collecting and preparing the core economic data, the environmental data, and indicators was not performed by either package. To assist data acquisition and transformation, the R language, which has many similarities in data science applications to Python, was used to create a set of interdependent R scripts, coupled with the *useeiopy* and *iomb* packages, to retrieve and process larger environmental datasets and assemble the simplified two-region USEEIO state models.

With a growing need for increased variety of USEEIO models for various applications, USEEIO was rebranded as a “family of models”, and received significant redesign in the modeling process. In response to the needs for more transparent and reproducible model build paths from data acquisition through to final output, as well as embedded model validation procedures, the *useeior* R package was developed, along with a versioning scheme [4] for USEEIO models. *useeior* simplifies and streamlines the modeling process and enables transparent and reproducible model construction. Users are provided with not only fundamental data and metadata to build default USEEIO models, but also great flexibility to customize models of their own. *useeior* replaces the *iomb/useeiopy* tools, and now serves as a core component of the US EPA’s USEEIO modeling framework, as it integrates the up-to-date IO tables prepared within itself with the environmental data generated by other tools within the framework, and then produces EEIO results in standard formats and software-ready LCI.

Designed and created with the principles of open-source software, *useeior* is continuously improved to be more comprehensive and up-to-date in a transparent and iterative way. Currently, *useeior* is capable of building USEEIO models that reflect the US national average economic and environmental conditions. Users should be aware of the limitations of using the national average to estimate environmental impacts of goods and services produced in a sub-national scope. To address user demand for sub-national models, regionalized versions of the model are being incorporated into *useeior* to enable construction of two-region (a US state and a rest of US region) USEEIO state models. Future improvements, such as physical hybrid models and linkage to global multi-regional input-output (MRIO) models are planned to facilitate additional extensions in *useeior*.

## Supplementary Material

Supplementary Material

## Figures and Tables

**Table 1.** Built-in models in *useeior* v1.0.0. All models are single-region USEEIO models, with the 50 states of the US modeled as one region. IO data used in all models are before BEA’s redefinition and in producer price.

Model Name	Description	Number of Sector	Number of Impact Category	IO Data Year	Environmental Data Years
USEEIOv2.0	A detail level commodity model with full life cycle inventory	405	23	2012	2010–2017
USEEIOv2.0-411	A detail level commodity model with waste sector disaggregation and full life cycle inventory	411 (404 + 7)	23	2012	2010–2017
USEEIOv2.0.1-411	A detail level commodity model with waste sector disaggregation and full life cycle inventory, including updated satellite tables with UUIDs	411 (404 + 7)	23	2012	2010–2017
USEEIOv2.1-422	A detail level commodity model with waste sector disaggregation and electricity sector aggregation and disaggregation	422 (403 + 19)	23	2012	2010–2017
USEEIOv2.0 GHG	A detail level commodity model with life cycle inventory of greenhouse gas (GHG)	405	1	2012	2016
USEEIOv2.0-i-GHG	A detail level industry model with life cycle inventory of greenhouse gas	405	1	2012	2016
USEEIOv2.0-s-GHG	A summary level commodity model with life cycle inventory of greenhouse gas	73	1	2012	2016
USEEIOv2.0-79-GHG	A summary level commodity model with waste sector disaggregation and life cycle inventory of greenhouse gas	79	1	2012	2016
USEEIOv2.0-is-GHG	A summary level industry model with life cycle inventory of greenhouse gas	71	1	2012	2016

**Table 2.** Contribution from top 5 flows to total acidification potential in the Utilities sector.

Flow	Contribution
Sulfur dioxide/emission/air/kg	56.1%
Nitrogen dioxide/emission/air/kg	40.0%
Ammonia/emission/air/kg	2.8%
Sulfuric acid/emission/air/kg	0.7%
Hydrofluoric acid/emission/air/kg	0.2%

**Table 3.** Contribution from top 5 sectors to direct freshwater withdrawals in the food and beverage and tobacco products sector.

Sector	Contribution
111CA/US—Farms	92.1%
22/US—Utilities	4.7%
311FT/US—Food and beverage and tobacco products	1.6%
113FF/US—Forestry, fishing, and related activities	0.9%
332/US—Fabricated metal products	0.1%

## Data Availability

Data used to demonstrate *useeior* are available as part of the *useeior* software package. Source code for *useeior* is available at https://github.com/usepa/useeior, (accessed on 14 March 2022).
