# Supplementary material for: useeior: An Open-Source R Package for Building and Using US Environmentally-Extended Input–Output Models
Source: Appl Sci (Basel). Author manuscript; Available in PMC 2022 Jun 8. (PMC9175389; doi:10.3390/app12094469)
Supplement: Supplementary Material [file NIHMS1808822-supplement-Supplementary_Material.pdf]

useeior: An open source R Package for Building and Using US Environmentally-Extended Input-Output Models

Supplementary Information

Mo Li*[1]

Wesley W. Ingwersen*[2]

Ben Young[3]

Jorge Vendries[4]

Catherine Birney[5]

This SI document demonstrates the complete process of building a USEEIO model with *useeior v1.0.0*.

S1 Download and load *useeior*

useeior v1.0.0 can be downloaded from GitHub upon successful installation of the `devtools` package. Loading *useeior* via the `library` function allows direct access to model building functions in *useeior*.

```
if (!"devtools" %in% installed.packages()) install.packages(devtools)
devtools::install_github("USEPA/useeior@v1.0.0")
library(useeior)
citation("useeior")
```

```
##
## To cite package 'useeior' in publications use:
##
##   Mo Li, Wesley Ingwersen, Ben Young, Jorge Vendries and Catherine
##   Birney (2021). useeior: USEEIO R modeling software. R package version
```

[1] General Dynamics Information Technology, Inc. *mo.li@gdit.com

[2] Office of Research and Development, US Environmental Protection Agency *ingwersen.wesley@epa.gov

[3] Eastern Research Group

[4] Eastern Research Group

[5] Office of Research and Development, US Environmental Protection Agency

```
##   1.0.0. https://github.com/USEPA/useeior
##
## A BibTeX entry for LaTeX users is
##
##   @Manual{,
##     title = {useeior: USEEIO R modeling software},
##     author = {Mo Li and Wesley Ingwersen and Ben Young and Jorge Vendries
and Catherine Birney},
##     year = {2021},
##     note = {R package version 1.0.0},
##     url = {https://github.com/USEPA/useeior},
##   }
```

It is recommended to first check the available models in *useeior*. If the desired model is already built-in with the package, it can be directly built with the `buildModel` function. Otherwise, a valid model configuration file (`.yml`) is required.

```
seeAvailableModels()
```

```
## [1] "USEEIOv2.0-411"
## [2] "USEEIOv2.0-79-GHG"
## [3] "USEEIOv2.0-GHG"
## [4] "USEEIOv2.0-i-GHG"
## [5] "USEEIOv2.0-is-GHG"
## [6] "USEEIOv2.0-s-GHG"
## [7] "USEEIOv2.0.1-411"
## [8] "USEEIOv2.0"
## [9] "USEEIOv2.1-422"
```

S2 Load USEEIOv2.0.1s model configuration

Here is an example of building a model that is not available in *useeior*. Declare the model to use as USEEIOv2.0.1s and the model configuration file to use as USEEIOv2.0.1s.yml.

```
modelname <- "USEEIOv2.0.1s"
configpaths <- "model_specs/USEEIOv2.0.1s.yml"
```

Below is a complete model configuration file of USEEIOv2.0.1s.

```
Model: "USEEIOv2.0.1s"
BaseIOSchema: 2012
BaseIOLevel: &BaseIOLevel "Summary"
IOYear: 2018 # Year for IO data
ModelRegionAcronyms: ["US"]
ModelType: "EEIO"
IODataSource: "BEA"
BasePriceType: "PRO" #producer
BasewithRedefinitions: FALSE
CommodityorIndustryType: "Commodity"
ScrapIncluded: FALSE
```

```yaml
DisaggregationSpecs: NULL

SatelliteTable:
  WAT:
    FullName: "Water withdrawals"
    Abbreviation: "WAT"
    StaticSource: TRUE
    StaticFile:
"flowsa/FlowBySector/Water_national_2015_m1_v1.0.0_c31cd44.parquet"
    FileLocation: "DataCommons"
    DataYears: [2015]
    Locations: ["US"]
    SectorListSource: "NAICS"
    SectorListYear: 2012
    SectorListLevel: "6"
    OriginalFlowSource: "FEDEFLv1.0.6"
    ScriptFunctionCall: "getFlowbySectorCollapsed" #function to call for
script
    ScriptFunctionParameters: null
    DataSources:
      USGS_NWIS_WU_2015:
        Title: "Water Use in the US"
        Author: "USGS"
        DataYear: 2015
        URL: "https://waterdata.usgs.gov/"
        Primary: TRUE
  CHAIR:
    FullName: "Criteria and Hazardous Air Emissions"
    Abbreviation: "CHAIR"
    StaticSource: TRUE
    StaticFile:
"flowsa/FlowBySector/CAP_HAP_national_2017_v0.3.1_f3cdf5b.parquet"
    FileLocation: "DataCommons"
    DataYears: [2017]
    Locations: ["US"]
    SectorListSource: "NAICS"
    SectorListYear: 2012
    SectorListLevel: "6"
    OriginalFlowSource: "FEDEFLv1.0.6"
    ScriptFunctionCall: "getFlowbySectorCollapsed" #function to call for
script
    ScriptFunctionParameters: null
    DataSources:
      EPA_NEI_2017:
        Title: "National Emissions Inventory"
        Author: "EPA"
        DataYear: 2017
        URL: "https://www.epa.gov/air-emissions-inventories/national-
emissions-inventory-nei"
        Primary: TRUE
```

```yaml
      EPA_TRI_2017:
        Title: "Toxic Release Inventory"
        Author: "EPA"
        DataYear: 2017
        URL: "https://www.epa.gov/toxics-release-inventory-tri-program"
        Primary: TRUE
  GRDREL:
    FullName: "Point source industrial releases to ground"
    Abbreviation: "GRDREL"
    StaticSource: TRUE
    StaticFile:
"flowsa/FlowBySector/GRDREL_national_2017_v0.3.1_f3cdf5b.parquet"
    FileLocation: "DataCommons"
    DataYears: [2017]
    Locations: ["US"]
    SectorListSource: "NAICS"
    SectorListYear: 2012
    SectorListLevel: "6"
    OriginalFlowSource: "FEDEFLv1.0.6"
    ScriptFunctionCall: "getFlowbySectorCollapsed" #function to call for
script
    ScriptFunctionParameters: null
    DataSources:
      EPA_TRI_2017:
        Title: "Toxic Release Inventory"
        Author: "EPA"
        DataYear: 2017
        URL: "https://www.epa.gov/toxics-release-inventory-tri-program"
        Primary: TRUE
  WATREL:
    FullName: "Point source releases to water"
    Abbreviation: "WATREL"
    StaticSource: TRUE
    StaticFile:
"flowsa/FlowBySector/TRI_DMR_national_2017_v0.3.1_f3cdf5b.parquet"
    FileLocation: "DataCommons"
    DataYears: [2017]
    Locations: ["US"]
    SectorListSource: "NAICS"
    SectorListYear: 2012
    SectorListLevel: "6"
    OriginalFlowSource: "FEDEFLv1.0.6"
    ScriptFunctionCall: "getFlowbySectorCollapsed" #function to call for
script
    ScriptFunctionParameters: null
    DataSources:
      EPA_TRI_2017:
        Title: "Toxic Release Inventory"
        Author: "EPA"
        DataYear: 2017
```

```
        URL: "https://www.epa.gov/toxics-release-inventory-tri-program"
        Primary: TRUE
      EPA_DMR_2017:
        Title: "Discharge Monitoring Report"
        Author: "EPA"
        DataYear: 2017
        URL: "https://echo.epa.gov/tools/data-downloads/icis-npdes-dmr-and-
limit-data-set"
        Primary: TRUE
  GHG:
    FullName: "Greenhouse Gases"
    Abbreviation: "GHG"
    StaticSource: TRUE
    StaticFile: "useeior/NGHGIAM_GHG_TotalsBySector_wUUIDs.csv"
    FileLocation: "DataCommons"
    DataYears: [2016]
    Locations: ["US"]
    SectorListSource: "BEA" # or, NAICS
    SectorListYear: 2012
    SectorListLevel: "Detail"
    OriginalFlowSource: "FEDEFLv1.0.6"
    DataSources:
      USEPA_GHG_2018:
        Title: "GHG Inventory"
        Author: "USEPA"
        DataYear: 2016
        URL: "https://www.epa.gov/ghgemissions/inventory-us-greenhouse-gas-
emissions-and-sinks-1990-2016"
        Primary: TRUE
  LAND:
    FullName: "Land use"
    Abbreviation: "LAND"
    StaticSource: TRUE
    StaticFile:
"flowsa/FlowBySector/Land_national_2012_v1.0.0_c31cd44.parquet"
    FileLocation: "DataCommons"
    DataYears: [2012]
    Locations: ["US"]
    SectorListSource: "NAICS"
    SectorListYear: 2012
    SectorListLevel: "6"
    OriginalFlowSource: "FEDEFLv1.0.6"
    ScriptFunctionCall: "getFlowbySectorCollapsed" #function to call for
script
    ScriptFunctionParameters: null
    DataSources:
      BLM_PLS:
        Title: "Public Land Statistics"
        Author: "BLM"
        DataYear: 2012
```

```
      URL: "https://www.blm.gov/about/data/public-land-statistics"
      Primary: FALSE
    EIA_CBECS_Land:
      Title: "Commercial Building Energy Consumption Survey"
      Author: "EIA"
      DataYear: 2012
      URL: "https://www.eia.gov/consumption/commercial/data/2012/"
      Primary: FALSE
    EIA_MECS_Land:
      Title: "Manufacturing Energy Consumption Survey"
      Author: "EIA"
      DataYear: 2014
      URL: "https://www.eia.gov/consumption/manufacturing/data/2014/"
      Primary: FALSE
    USDA_ERS_MLU:
      Title: "Major Uses of Land in the United States"
      Author: "USDA"
      DataYear: 2012
      URL: "https://www.ers.usda.gov/data-products/major-land-uses/"
      Primary: TRUE
MINE:
  FullName: "Mineral extraction"
  Abbreviation: "MINE"
  StaticSource: TRUE
  StaticFile: "useeior/USEEIOv1.1_MINE_TotalsBySector_FEDEFL_wUUIDs.csv"
  FileLocation: "DataCommons"
  DataYears: [2014]
  Locations: ["US"]
  SectorListSource: "BEA"
  SectorListYear: 2007
  SectorListLevel: "Detail"
  OriginalFlowSource: "FEDEFLv1.0.6"
  DataSources:
     USGS_MCS:
      Title: "Mineral Commodity Summary"
      Author: "USGS"
      DataYear: 2014
      URL: "https://www.usgs.gov/centers/nmic/mineral-commodity-summaries"
      Primary: TRUE
ENERGY:
  FullName: "Energy extraction"
  Abbreviation: "ENERGY"
  StaticSource: TRUE
  StaticFile: "useeior/USEEIOv1.1_Energy_TotalsBySector_FEDEFL_wUUIDs.csv"
  FileLocation: "DataCommons"
  DataYears: [2014]
  Locations: ["US"]
  SectorListSource: "BEA"
  SectorListYear: 2007
  SectorListLevel: "Detail"
```

```yaml
    OriginalFlowSource: "FEDEFLv1.0.6"
    DataSources:
      EIA_MER:
        Title: "Monthly Energy Review"
        Author: "EIA"
        DataYear: 2014
        URL: "http://www.eia.gov/totalenergy/data/monthly/"
        Primary: TRUE
      EIA_923:
        Title: "Form EIA-923 Detailed"
        Author: "EIA"
        DataYear: 2014
        URL: "https://www.eia.gov/electricity/data/eia923/"
        Primary: TRUE
  NPAG:
    FullName: "Nitrogen and Phosphorus Releases from Agriculture"
    Abbreviation: "NPAG"
    StaticSource: TRUE
    StaticFile: "useeior/USEEIOv1.1_NPAG_TotalsBySector_wUUIDs.csv"
    FileLocation: "DataCommons"
    DataYears: [2013, 2015]
    Locations: ["US"]
    SectorListSource: "BEA"
    SectorListYear: 2007
    SectorListLevel: "Detail"
    OriginalFlowSource: "FEDEFLv1.0.6"
    DataSources:
      USDA_CUS_Corn_Potatoes:
        Title: "Chemical Use Survey - Corn and Potatoes"
        Author: "USDA"
        DataYear: 2014
        URL: "https://www.nass.usda.gov/Data_and_Statistics/Pre-
Defined_Queries/2014_Corn_and_Potatoes/"
        Primary: FALSE
      USDA_CUS_Cotton:
        Title: "Chemical Use Survey - Cotton"
        Author: "USDA"
        DataYear: 2010
        URL: "https://www.nass.usda.gov/Data_and_Statistics/Pre-
Defined_Queries/2010_Corn_Upland_Cotton_Fall_Potatoes/"
        Primary: FALSE
      USDA_CUS_Fruit:
        Title: "Chemical Use Survey - Fruit"
        Author: "USDA"
        DataYear: 2011
        URL: "https://www.nass.usda.gov/Data_and_Statistics/Pre-
Defined_Queries/2011_Fruit_Chem_Usage/"
        Primary: FALSE
      USDA_CUS_Peanuts_Rice:
        Title: "Chemical Use Survey - Peanuts and Rice"
```

```
        Author: "USDA"
        DataYear: 2013
        URL: "https://www.nass.usda.gov/Data_and_Statistics/Pre-
Defined_Queries/2013_Peanuts_and_Rice/"
        Primary: FALSE
      USDA_CUS_Soybeans_Wheat:
        Title: "Chemical Use Survey - Soybeans and Wheat"
        Author: "USDA"
        DataYear: 2012
        URL: "https://www.nass.usda.gov/Data_and_Statistics/Pre-
Defined_Queries/2012_Soybeans_and_Wheat/"
        Primary: FALSE
      USDA_CUS_Vegetables:
        Title: "Chemical Use Survey - Vegetables"
        Author: "USDA"
        DataYear: 2014
        URL: "https://www.nass.usda.gov/Data_and_Statistics/Pre-
Defined_Queries/2014_Vegetables/"
        Primary: FALSE
  PEST:
    FullName: "Pesticide releases"
    Abbreviation: "PEST"
    StaticSource: TRUE
    StaticFile: "useeior/USEEIOv1.1_PEST_TotalsBySector_wUUIDs.csv"
    FileLocation: "DataCommons"
    DataYears: [2004, 2005, 2010, 2011, 2012, 2013, 2014]
    Locations: ["US"]
    SectorListSource: "BEA"
    SectorListYear: 2007
    SectorListLevel: "Detail"
    OriginalFlowSource: "FEDEFLv1.0.6"
    DataSources:
      USDA_CUS_Corn_Potatoes:
        Title: "Chemical Use Survey - Corn and Potatoes"
        Author: "USDA"
        DataYear: 2014
        URL: "https://www.nass.usda.gov/Data_and_Statistics/Pre-
Defined_Queries/2014_Corn_and_Potatoes/"
        Primary: FALSE
      USDA_CUS_Cotton:
        Title: "Chemical Use Survey - Cotton"
        Author: "USDA"
        DataYear: 2010
        URL: "https://www.nass.usda.gov/Data_and_Statistics/Pre-
Defined_Queries/2010_Corn_Upland_Cotton_Fall_Potatoes/"
        Primary: FALSE
      USDA_CUS_Fruit:
        Title: "Chemical Use Survey - Fruit"
        Author: "USDA"
        DataYear: 2011
```

```
        URL: "https://www.nass.usda.gov/Data_and_Statistics/Pre-
Defined_Queries/2011_Fruit_Chem_Usage/"
        Primary: FALSE
      USDA_CUS_Peanuts_Rice:
        Title: "Chemical Use Survey - Peanuts and Rice"
        Author: "USDA"
        DataYear: 2013
        URL: "https://www.nass.usda.gov/Data_and_Statistics/Pre-
Defined_Queries/2013_Peanuts_and_Rice/"
        Primary: FALSE
      USDA_CUS_Soybeans_Wheat:
        Title: "Chemical Use Survey - Soybeans and Wheat"
        Author: "USDA"
        DataYear: 2012
        URL: "https://www.nass.usda.gov/Data_and_Statistics/Pre-
Defined_Queries/2012_Soybeans_and_Wheat/"
        Primary: FALSE
      USDA_CUS_Vegetables:
        Title: "Chemical Use Survey - Vegetables"
        Author: "USDA"
        DataYear: 2014
        URL: "https://www.nass.usda.gov/Data_and_Statistics/Pre-
Defined_Queries/2014_Vegetables/"
        Primary: FALSE
  CNHW:
    FullName: "Commercial non-hazardous waste excluding construction
activities"
    Abbreviation: "CNHW"
    StaticSource: TRUE
    StaticFile: "useeior/USEEIOv1.1_CNHW_TotalsBySector.csv"
    FileLocation: "DataCommons"
    DataYears: [2015]
    Locations: ["US"]
    SectorListSource: "BEA"
    SectorListYear: 2007
    SectorListLevel: "Detail"
    OriginalFlowSource: "Waste Characterization Study 2014"
    DataSources:
      CalRecycle_CWCS:
        Title: "2014 Generator-Based Characterization of Commercial Sector"
        Author: "CalRecycle"
        DataYear: 2014
        URL:
"https://www2.calrecycle.ca.gov/WasteCharacterization/PubExtracts/2014/GenSum
mary.pdf"
        Primary: TRUE
  CNHWC:
    FullName: "Commercial non-hazardous waste from construction activities"
    Abbreviation: "CNHWC"
    StaticSource: TRUE
```

```yaml
    StaticFile: "useeior/USEEIOv1.1_CNHWC_TotalsBySector.csv"
    FileLocation: "DataCommons"
    DataYears: [2014]
    Locations: ["US"]
    SectorListSource: "BEA"
    SectorListYear: 2007
    SectorListLevel: "Detail"
    OriginalFlowSource: "CDDPath"
    DataSources:
      USEPA_FF_2014:
        Title: "Advancing Sustainable Materials Management: 2014 Fact Sheet"
        Author: "USEPA"
        DataYear: 2014
        URL: "https://www.epa.gov/sites/production/files/2016-
11/documents/2014_smmfactsheet_508.pdf"
        Primary: TRUE
  CRHW:
    FullName: "Commercial RCRA-defined hazardous waste"
    Abbreviation: "CRHW"
    StaticSource: TRUE
    StaticFile:
"flowsa/FlowBySector/CRHW_national_2017_v0.3.1_f3cdf5b.parquet"
    FileLocation: "DataCommons"
    DataYears: [2017]
    Locations: ["US"]
    SectorListSource: "NAICS"
    SectorListYear: 2012
    SectorListLevel: "6"
    ScriptFunctionCall: "getFlowbySectorCollapsed" #function to call for
script
    ScriptFunctionParameters: null
    OriginalFlowSource: "RCRAInfo"
    DataSources:
      USEPA_RCRAInfo_2017:
        Title: "National Biennial RCRA Hazardous Waste Report"
        Author: "USEPA"
        DataYear: 2017
        URL:
"https://rcrapublic.epa.gov/rcrainfoweb/action/modules/br/main/broverview"
        Primary: TRUE
  EMP:
    FullName: "Employment"
    Abbreviation: "EMP"
    StaticSource: TRUE
    StaticFile:
"flowsa/FlowBySector/Employment_national_2017_v0.3.1_f3cdf5b.parquet"
    FileLocation: "DataCommons"
    DataYears: [2017]
    Locations: ["US"]
    SectorListSource: "NAICS"
```

```
    SectorListYear: 2012
    SectorListLevel: "6"
    ScriptFunctionCall: "getFlowbySectorCollapsed" #function to call for
script
    ScriptFunctionParameters: null
    OriginalFlowSource: "National Employment Matrix"
    DataSources:
      BLS_QCEW_2017:
        Title: "Quarterly Census of Employment and Wages"
        Author: "BLS"
        DataYear: 2017
        URL: "https://www.bls.gov/cew/"
        Primary: TRUE
  VADD:
    FullName: "Value Added"
    Abbreviation: "VADD"
    StaticSource: FALSE
    StaticFile: null
    FileLocation: None
    DataYears: [2012]
    Locations: ["US"]
    SectorListSource: "BEA"
    SectorListYear: 2012
    SectorListLevel: *BaseIOLevel
    OriginalFlowSource: "Input-Output Tables"
    ScriptFunctionCall: "getValueAddedTotalsbySector" #function to call for
script
    ScriptFunctionParameters: ["model"] #list of parameters
    DataSources:
     BEA_USE:
       Title: "Detail Use Before Redefinitions"
       Author: "BEA"
       DataYear: 2012
       URL:
"https://apps.bea.gov//industry/iTables%20Static%20Files/AllTablesIO.zip"
       Primary: TRUE

Indicators:
  GreenhouseGases:
    Name: "Greenhouse Gases"
    Code: "GHG"
    Group: "Impact Potential"
    Unit: "kg CO2 eq"
    SimpleUnit: "Kilograms Carbon Dioxide (CO2)"
    SimpleName: "Greenhouse Gases"
    StaticSource: TRUE
    StaticFile: "lciafmt/traci/TRACI_2.1_v1.0.0_5555779.parquet"
    FileLocation: "DataCommons"
    ScriptFunctionCall: "getImpactMethod" #function to call for script
    ScriptFunctionParameters:
```

```
      indicators: ["Global warming"]
    DataSources:
      USEPA_TRACI_2.1:
        Title: "TRACI 2.1"
        Author: "USEPA"
        DataYear: NA
        URL: "https://www.epa.gov/chemical-research/tool-reduction-and-
assessment-chemicals-and-other-environmental-impacts-traci"
        Primary: TRUE
  Acidification:
    Name: "Acidification Potential"
    Code: "ACID"
    Group: "Impact Potential"
    Unit: "kg SO2 eq"
    SimpleUnit: "Kilograms Sulphur Dioxide (SO2)"
    SimpleName: "Acid Rain"
    StaticSource: TRUE
    StaticFile: "lciafmt/traci/TRACI_2.1_v1.0.0_5555779.parquet"
    FileLocation: "DataCommons"
    ScriptFunctionCall: "getImpactMethod" #function to call for script
    ScriptFunctionParameters:
      indicators: ["Acidification"]
    DataSources:
      USEPA_TRACI_2.1:
        Title: "TRACI 2.1"
        Author: "USEPA"
        DataYear: NA
        URL: "https://www.epa.gov/chemical-research/tool-reduction-and-
assessment-chemicals-and-other-environmental-impacts-traci"
        Primary: TRUE
  Eutrophication:
    Name: "Eutrophication Potential"
    Code: "EUTR"
    Group: "Impact Potential"
    Unit: "kg N eq"
    SimpleUnit: "Kilograms Nitrogen (N)"
    SimpleName: "Water Eutrophication"
    StaticSource: TRUE
    StaticFile: "lciafmt/traci/TRACI_2.1_v1.0.0_5555779.parquet"
    FileLocation: "DataCommons"
    ScriptFunctionCall: "getImpactMethod" #function to call for script
    ScriptFunctionParameters:
      indicators: ["Eutrophication"]
    DataSources:
      USEPA_TRACI_2.1:
        Title: "TRACI 2.1"
        Author: "USEPA"
        DataYear: NA
        URL: "https://www.epa.gov/chemical-research/tool-reduction-and-
assessment-chemicals-and-other-environmental-impacts-traci"
```

```yaml
        Primary: TRUE
  FreshwaterEcotox:
    Name: "Freshwater Ecotoxicity Potential"
    Code: "ETOX"
    Group: "Impact Potential"
    Unit: "CTUe"
    SimpleUnit: "Comparative Toxic Unit for Ecosystem (CTUe)"
    SimpleName: "Freshwater Ecotoxicity"
    StaticSource: TRUE
    StaticFile: "lciafmt/traci/TRACI_2.1_v1.0.0_5555779.parquet"
    FileLocation: "DataCommons"
    ScriptFunctionCall: "getImpactMethod" #function to call for script
    ScriptFunctionParameters:
      indicators: ["Freshwater ecotoxicity"]
    DataSources:
      USEPA_TRACI_2.1:
        Title: "TRACI 2.1"
        Author: "USEPA"
        DataYear: NA
        URL: "https://www.epa.gov/chemical-research/tool-reduction-and-
assessment-chemicals-and-other-environmental-impacts-traci"
        Primary: TRUE
  HumanHealthCancer:
    Name: "Human Health - Cancer"
    Code: "HCAN"
    Group: "Impact Potential"
    Unit: "CTUh"
    SimpleUnit: "Comparative Toxic Unit for Humans (CTUh)"
    SimpleName: "Cancer Disease"
    StaticSource: TRUE
    StaticFile: "lciafmt/traci/TRACI_2.1_v1.0.0_5555779.parquet"
    FileLocation: "DataCommons"
    ScriptFunctionCall: "getImpactMethod" #function to call for script
    ScriptFunctionParameters:
      indicators: ["Human health - cancer"]
    DataSources:
      USEPA_TRACI_2.1:
        Title: "TRACI 2.1"
        Author: "USEPA"
        DataYear: NA
        URL: "https://www.epa.gov/chemical-research/tool-reduction-and-
assessment-chemicals-and-other-environmental-impacts-traci"
        Primary: TRUE
  HumanHealthNonCancer:
    Name: "Human Health - Noncancer"
    Code: "HNCN"
    Group: "Impact Potential"
    Unit: "CTUh"
    SimpleUnit: "Comparative Toxic Unit for Humans (CTUh)"
    SimpleName: "Noncancer Disease"
```

```
    StaticSource: TRUE
    StaticFile: "lciafmt/traci/TRACI_2.1_v1.0.0_5555779.parquet"
    FileLocation: "DataCommons"
    ScriptFunctionCall: "getImpactMethod" #function to call for script
    ScriptFunctionParameters:
      indicators: ["Human health - non-cancer"]
    DataSources:
      USEPA_TRACI_2.1:
        Title: "TRACI 2.1"
        Author: "USEPA"
        DataYear: NA
        URL: "https://www.epa.gov/chemical-research/tool-reduction-and-
assessment-chemicals-and-other-environmental-impacts-traci"
        Primary: TRUE
  HumanHealthToxicity:
    Name: "Human Health Toxicity"
    Code: "HTOX"
    Group: "Impact Potential"
    Unit: "CTUh"
    SimpleUnit: "Comparative Toxic Unit for Humans (CTUh)"
    SimpleName: "Toxic to Humans"
    StaticSource: TRUE
    StaticFile: "lciafmt/traci/TRACI_2.1_v1.0.0_5555779.parquet"
    FileLocation: "DataCommons"
    ScriptFunctionCall: "getCombinedImpactMethods" #function to call for
script
    ScriptFunctionParameters:
      indicators: ["Human health - cancer", "Human health - non-cancer"]
    DataSources:
      USEEIO_LCIA_Aggregation:
        Title: "Aggregation of HNCN and HCAN"
        Author: NA
        DataYear: NA
        URL: NA
        Primary: TRUE
  HumanHealthRespEffects:
    Name: "Human Health - Respiratory Effects"
    Code: "HRSP"
    Group: "Impact Potential"
    Unit: "kg PM2.5 eq"
    SimpleUnit: "Kilograms Particulate Matter"
    SimpleName: "Respiratory Effects"
    StaticSource: TRUE
    StaticFile: "lciafmt/traci/TRACI_2.1_v1.0.0_5555779.parquet"
    FileLocation: "DataCommons"
    ScriptFunctionCall: "getImpactMethod" #function to call for script
    ScriptFunctionParameters:
      indicators: ["Human health - particulate matter"]
    DataSources:
      USEPA_TRACI_2.1:
```

```
        Title: "TRACI 2.1"
        Author: "USEPA"
        URL: "https://www.epa.gov/chemical-research/tool-reduction-and-
assessment-chemicals-and-other-environmental-impacts-traci"
        Primary: TRUE
  Ozone:
    Name: "Ozone Depletion"
    Code: "OZON"
    Group: "Impact Potential"
    Unit: "kg CFC-11 eq"
    SimpleUnit: "Kilograms ChloroFluoroCarbon-11"
    SimpleName: "Ozone Depletion"
    StaticSource: TRUE
    StaticFile: "lciafmt/traci/TRACI_2.1_v1.0.0_5555779.parquet"
    FileLocation: "DataCommons"
    ScriptFunctionCall: "getImpactMethod" #function to call for script
    ScriptFunctionParameters:
      indicators: ["Ozone depletion"]
    DataSources:
      USEPA_TRACI_2.1:
        Title: "TRACI 2.1"
        Author: "USEPA"
        DataYear: NA
        URL: "https://www.epa.gov/chemical-research/tool-reduction-and-
assessment-chemicals-and-other-environmental-impacts-traci"
        Primary: TRUE
  Smog:
    Name: "Smog Formation Potential"
    Code: "SMOG"
    Group: "Impact Potential"
    Unit: "kg O3 eq"
    SimpleUnit: "Kilograms Ozone (O3)"
    SimpleName: "Smog Formation"
    StaticSource: TRUE
    StaticFile: "lciafmt/traci/TRACI_2.1_v1.0.0_5555779.parquet"
    FileLocation: "DataCommons"
    ScriptFunctionCall: "getImpactMethod" #function to call for script
    ScriptFunctionParameters:
      indicators: ["Smog formation"]
    DataSources:
      USEPA_TRACI_2.1:
        Title: "TRACI 2.1"
        Author: "USEPA"
        DataYear: NA
        URL: "https://www.epa.gov/chemical-research/tool-reduction-and-
assessment-chemicals-and-other-environmental-impacts-traci"
        Primary: TRUE
  freshwater_withdrawal:
    Name: "Freshwater withdrawals"
    Code: "WATR"
```

```yaml
    Group: "Resource Use"
    Unit: "kg"
    SimpleUnit: "Kilograms"
    SimpleName: "Water Use"
    StaticSource: TRUE
    StaticFile: "lciafmt/fedefl/FEDEFL_Inventory_v1.0.0_5555779.parquet"
    FileLocation: "DataCommons"
    ScriptFunctionCall: "getImpactMethod" #function to call for script
    ScriptFunctionParameters:
      indicators: ["freshwater_resources"]
    DataSources:
      LCIAformatter:
        Title: "LCIAformatter FEDEFL Inventory Methods"
        Author: "USEPA"
        DataYear: 2020
        URL: "https://github.com/USEPA/LCIAformatter"
        Primary: TRUE
  land_use:
    Name: "Land use"
    Code: "LAND"
    Group: "Resource Use"
    Unit: "m2*yr"
    SimpleUnit: "Square Meters per Year"
    SimpleName: "Land Use"
    StaticSource: TRUE
    StaticFile: "lciafmt/fedefl/FEDEFL_Inventory_v1.0.0_5555779.parquet"
    FileLocation: "DataCommons"
    ScriptFunctionCall: "getImpactMethod" #function to call for script
    ScriptFunctionParameters:
      indicators: ["land_use"]
    DataSources:
      LCIAformatter:
        Title: "LCIAformatter FEDEFL Inventory Methods"
        Author: "USEPA"
        DataYear: 2020
        URL: "https://github.com/USEPA/LCIAformatter"
        Primary: TRUE
  HAP:
    Name: "Hazardous Air Pollutants"
    Code: "HAPS"
    Group: "Chemical Releases"
    Unit: "kg"
    SimpleUnit: "Kilograms"
    SimpleName: "Hazardous Air Pollutants"
    StaticSource: TRUE
    StaticFile: "lciafmt/fedefl/FEDEFL_Inventory_v1.0.0_5555779.parquet"
    FileLocation: "DataCommons"
    ScriptFunctionCall: "getImpactMethod" #function to call for script
    ScriptFunctionParameters:
      indicators: ["HAP"]
```

```yaml
  DataSources:
    LCIAformatter:
      Title: "LCIAformatter FEDEFL Inventory Methods"
      Author: "USEPA"
      DataYear: 2020
      URL: "https://github.com/USEPA/LCIAformatter"
      Primary: TRUE
Pesticides:
  Name: "Pesticides"
  Code: "PEST"
  Group: "Chemical Releases"
  Unit: "kg"
  SimpleUnit: "Kilograms"
  SimpleName: "Pesticides"
  StaticSource: TRUE
  StaticFile: "lciafmt/fedefl/FEDEFL_Inventory_v1.0.0_5555779.parquet"
  FileLocation: "DataCommons"
  ScriptFunctionCall: "getImpactMethod" #function to call for script
  ScriptFunctionParameters:
    indicators: ["USDA_CUS_pesticides"]
  DataSources:
    LCIAformatter:
      Title: "LCIAformatter FEDEFL Inventory Methods"
      Author: "USEPA"
      DataYear: 2020
      URL: "https://github.com/USEPA/LCIAformatter"
      Primary: TRUE
NonRenewableEnergy:
  Name: "Nonrenewable Energy Use"
  Code: "NNRG"
  Group: "Resource Use"
  Unit: "MJ"
  SimpleUnit: "Megajoules (MJ)"
  SimpleName: "Nonrenewable Energy Use"
  StaticSource: TRUE
  FileLocation: "DataCommons"
  StaticFile: "lciafmt/fedefl/FEDEFL_Inventory_v1.0.0_5555779.parquet"
  ScriptFunctionCall: "getImpactMethod" #function to call for script
  ScriptFunctionParameters:
    indicators: ["nonrenewable_energy"]
  DataSources:
    LCIAformatter:
      Title: "LCIAformatter FEDEFL Inventory Methods"
      Author: "USEPA"
      DataYear: 2020
      URL: "https://github.com/USEPA/LCIAformatter"
      Primary: TRUE
RenewableEnergy:
  Name: "Renewable Energy Use"
  Code: "RNRG"
```

```yaml
    Group: "Resource Use"
    Unit: "MJ"
    SimpleUnit: "Megajoules (MJ)"
    SimpleName: "Renewable Energy Use"
    StaticSource: TRUE
    StaticFile: "lciafmt/fedefl/FEDEFL_Inventory_v1.0.0_5555779.parquet"
    FileLocation: "DataCommons"
    ScriptFunctionCall: "getImpactMethod" #function to call for script
    ScriptFunctionParameters:
      indicators: ["renewable_energy"]
    DataSources:
      LCIAformatter:
        Title: "LCIAformatter FEDEFL Inventory Methods"
        Author: "USEPA"
        DataYear: 2020
        URL: "https://github.com/USEPA/LCIAformatter"
        Primary: TRUE
  Energy:
    Name: "Energy Use"
    Code: "ENRG"
    Group: "Resource Use"
    Unit: "MJ"
    SimpleUnit: "Megajoules (MJ)"
    SimpleName: "Energy Use"
    StaticSource: TRUE
    StaticFile: "lciafmt/fedefl/FEDEFL_Inventory_v1.0.0_5555779.parquet"
    FileLocation: "DataCommons"
    ScriptFunctionCall: "getImpactMethod" #function to call for script
    ScriptFunctionParameters:
      indicators: ["energy"]
    DataSources:
      LCIAformatter:
        Title: "LCIAformatter FEDEFL Inventory Methods"
        Author: "USEPA"
        DataYear: 2020
        URL: "https://github.com/USEPA/LCIAformatter"
        Primary: TRUE
  MineralsMetals:
    Name: "Minerals and Metals Use"
    Code: "MNRL"
    Group: "Resource Use"
    Unit: "kg"
    SimpleUnit: "Kilograms"
    SimpleName: "Minerals and Metals Use"
    StaticSource: TRUE
    StaticFile: "lciafmt/fedefl/FEDEFL_Inventory_v1.0.0_5555779.parquet"
    FileLocation: "DataCommons"
    ScriptFunctionCall: "getImpactMethod" #function to call for script
    ScriptFunctionParameters:
      indicators: ["USGS_mineral_resources"]
```

```
    DataSources:
      LCIAformatter:
        Title: "LCIAformatter FEDEFL Inventory Methods"
        Author: "USEPA"
        DataYear: 2020
        URL: "https://github.com/USEPA/LCIAformatter"
        Primary: TRUE
  ValueAdded:
    Name: "Value Added"
    Code: "VADD"
    Group: "Economic & Social"
    Unit: "$"
    SimpleUnit: "US Dollars ($)"
    SimpleName: "Value Added"
    StaticSource: TRUE
    StaticFile: "USEEIO_LCIA_Factors.csv"
    FileLocation: "useeior"
    DataSources:
      ingwersen_useeiov11_2017:
        Title: "USEEIOv1.1 - Elementary Flows and Life Cycle Impact
Assessment (LCIA) Characterization Factors"
        Author: "USEPA"
        DataYear: 2020
        URL: "http://doi.org/10.23719/1368541"
        Primary: TRUE
  Jobs:
    Name: "Jobs Supported"
    Code: "JOBS"
    Group: "Economic & Social"
    Unit: "jobs"
    SimpleUnit: "Employees"
    SimpleName: "Jobs Supported"
    StaticSource: TRUE
    StaticFile: "USEEIO_LCIA_Factors.csv"
    FileLocation: "useeior"
    DataSources:
      ingwersen_useeiov11_2017:
        Title: "USEEIOv1.1 - Elementary Flows and Life Cycle Impact
Assessment (LCIA) Characterization Factors"
        Author: "USEPA"
        DataYear: 2020
        URL: "http://doi.org/10.23719/1368541"
        Primary: TRUE
  RCRAHazWaste:
    Name: "Commercial RCRA Hazardous Waste"
    Code: "CRHW"
    Group: "Waste Generated"
    Unit: "kg"
    SimpleUnit: "Kilograms"
    SimpleName: "Hazardous Waste"
```

```
    StaticSource: TRUE
    StaticFile: "USEEIO_LCIA_Factors.csv"
    FileLocation: "useeior"
    DataSources:
      meyer_analyzing_2020:
        Title: "Analyzing economy-scale solid waste generation using the
United States environmentally-extended input-output model"
        Author: "USEPA"
        DataYear: 2020
        URL: "http://doi.org/10.1016/j.resconrec.2020.104795"
        Primary: TRUE
  MunicipalWaste:
    Name: "Commercial Municipal Solid Waste"
    Code: "CMSW"
    Group: "Waste Generated"
    Unit: "kg"
    SimpleUnit: "Kilograms"
    SimpleName: "Municipal Solid Waste"
    StaticSource: TRUE
    StaticFile: "USEEIO_LCIA_Factors.csv"
    FileLocation: "useeior"
    DataSources:
      meyer_analyzing_2020:
        Title: "Analyzing economy-scale solid waste generation using the
United States environmentally-extended input-output model"
        Author: "USEPA"
        DataYear: 2020
        URL: "http://doi.org/10.1016/j.resconrec.2020.104795"
        Primary: TRUE
  ConstructionDebris:
    Name: "Commercial Construction and Demolition Debris"
    Code: "CCDD"
    Group: "Waste Generated"
    Unit: "kg"
    SimpleUnit: "Kilograms"
    SimpleName: "Construction Debris"
    StaticSource: TRUE
    StaticFile: "USEEIO_LCIA_Factors.csv"
    FileLocation: "useeior"
    DataSources:
      meyer_analyzing_2020:
        Title: "Analyzing economy-scale solid waste generation using the
United States environmentally-extended input-output model"
        Author: "USEPA"
        DataYear: 2020
        URL: "http://doi.org/10.1016/j.resconrec.2020.104795"
        Primary: TRUE

DemandVectors:
    DefaultDemand: "DefaultDemandVectors" # Name of default demand vectors
```

```
yml file
# Additional demand vectors beyond useeior defaults
```

Because USEEIOv2.0.1s builds with default demand vectors, a `DefaultDemandVectors.yml` file specifying the default demand vector configuration is required to accompany the model configuration file in the same `/model_specs` folder. Below is a complete configuration file of the default demand vector.

```
CompleteProduction:
      Type: "Production"
      System: "Complete"
DomesticProduction:
      Type: "Production"
      System: "Domestic"
CompleteConsumption:
      Type: "Consumption"
      System: "Complete"
DomesticConsumption:
      Type: "Consumption"
      System: "Domestic"
```

S3 Build model

With valid model configuration and related files, USEEIOv2.0.1s can be built with the `buildModel` function. Each step in the process is logged with date and time stamp for transparency. A 'Model build complete' message will appear, once the model is successfully built.

```
model <- buildModel(modelname, configpaths)
```

```
## 2022-01-31 15:18:25 INFO::Begin model initialization...
## 2022-01-31 15:18:25 INFO::Initializing IO tables...
## 2022-01-31 15:18:25 INFO::Initializing Gross Output tables...
## 2022-01-31 15:18:26 INFO::Initializing Chain Price Index tables...
## 2022-01-31 15:18:26 INFO::Initializing model satellite tables...
## 2022-01-31 15:18:26 INFO::Loading Water withdrawals flows from
DataCommons...
## 2022-01-31 15:18:26 INFO::Loading Criteria and Hazardous Air Emissions
flows from DataCommons...
## 2022-01-31 15:18:33 INFO::Loading Point source industrial releases to
ground flows from DataCommons...
## 2022-01-31 15:18:33 INFO::Loading Point source releases to water flows
from DataCommons...
## 2022-01-31 15:18:34 INFO::Loading Greenhouse Gases flows from
DataCommons...
## 2022-01-31 15:18:34 INFO::Loading Land use flows from DataCommons...
## 2022-01-31 15:18:34 INFO::Loading Mineral extraction flows from
DataCommons...
## 2022-01-31 15:18:34 INFO::Loading Energy extraction flows from
```

```
DataCommons...
## 2022-01-31 15:18:34 INFO::Loading Nitrogen and Phosphorus Releases from
Agriculture flows from DataCommons...
## 2022-01-31 15:18:34 INFO::Loading Pesticide releases flows from
DataCommons...
## 2022-01-31 15:18:34 INFO::Loading Commercial non-hazardous waste excluding
construction activities flows from DataCommons...
## 2022-01-31 15:18:35 INFO::Loading Commercial non-hazardous waste from
construction activities flows from DataCommons...
## 2022-01-31 15:18:35 INFO::Loading Commercial RCRA-defined hazardous waste
flows from DataCommons...
## 2022-01-31 15:18:37 INFO::Loading Employment flows from DataCommons...
## 2022-01-31 15:18:37 INFO::Generating Value Added flows...
## 2022-01-31 15:18:37 INFO::Initializing model indicators...
## 2022-01-31 15:18:37 INFO::Getting Greenhouse Gases indicator from
DataCommons...
## 2022-01-31 15:18:37 INFO::Getting Acidification Potential indicator from
DataCommons...
## 2022-01-31 15:18:37 INFO::Getting Eutrophication Potential indicator from
DataCommons...
## 2022-01-31 15:18:37 INFO::Getting Freshwater Ecotoxicity Potential
indicator from DataCommons...
## 2022-01-31 15:18:43 INFO::Getting Human Health - Cancer indicator from
DataCommons...
## 2022-01-31 15:18:44 INFO::Getting Human Health - Noncancer indicator from
DataCommons...
## 2022-01-31 15:18:45 INFO::Getting Human Health Toxicity indicator from
DataCommons...
## 2022-01-31 15:18:47 INFO::Getting Human Health - Respiratory Effects
indicator from DataCommons...
## 2022-01-31 15:18:48 INFO::Getting Ozone Depletion indicator from
DataCommons...
## 2022-01-31 15:18:48 INFO::Getting Smog Formation Potential indicator from
DataCommons...
## 2022-01-31 15:18:49 INFO::Getting Freshwater withdrawals indicator from
DataCommons...
## 2022-01-31 15:18:49 INFO::Getting Land use indicator from DataCommons...
## 2022-01-31 15:18:49 INFO::Getting Hazardous Air Pollutants indicator from
DataCommons...
## 2022-01-31 15:18:49 INFO::Getting Pesticides indicator from DataCommons...
## 2022-01-31 15:18:49 INFO::Getting Nonrenewable Energy Use indicator from
DataCommons...
## 2022-01-31 15:18:49 INFO::Getting Renewable Energy Use indicator from
DataCommons...
## 2022-01-31 15:18:50 INFO::Getting Energy Use indicator from DataCommons...
## 2022-01-31 15:18:50 INFO::Getting Minerals and Metals Use indicator from
DataCommons...
## 2022-01-31 15:18:50 INFO::Getting Value Added indicator from useeior...
## 2022-01-31 15:18:50 INFO::Getting Jobs Supported indicator from useeior...
## 2022-01-31 15:18:50 INFO::Getting Commercial RCRA Hazardous Waste
```

```
indicator from useeior...
## 2022-01-31 15:18:50 INFO::Getting Commercial Municipal Solid Waste
indicator from useeior...
## 2022-01-31 15:18:50 INFO::Getting Commercial Construction and Demolition
Debris indicator from useeior...
## 2022-01-31 15:18:50 INFO::Loading demand vectors ...
## 2022-01-31 15:18:50 INFO::Loading CompleteProduction demand vector...
## 2022-01-31 15:18:50 INFO::Loading DomesticProduction demand vector...
## 2022-01-31 15:18:50 INFO::Loading CompleteConsumption demand vector...
## 2022-01-31 15:18:50 INFO::Loading DomesticConsumption demand vector...
## 2022-01-31 15:18:51 INFO::Building commodity-by-commodity A matrix (direct
requirements)...
## 2022-01-31 15:18:51 INFO::Building commodity-by-commodity A_d matrix
(domestic direct requirements)...
## 2022-01-31 15:18:51 INFO::Calculating L matrix (total requirements)...
## 2022-01-31 15:18:51 INFO::Calculating L_d matrix (domestic total
requirements)...
## 2022-01-31 15:18:51 INFO::Building B matrix (direct emissions and resource
use per dollar)...
## 2022-01-31 15:18:52 INFO::Building C matrix (characterization factors for
model indicators)...
## 2022-01-31 15:18:57 INFO::Calculating D matrix (direct environmental
impacts per dollar)...
## 2022-01-31 15:18:57 INFO::Calculating M matrix (total emissions and
resource use per dollar)...
## 2022-01-31 15:18:57 INFO::Calculating M_d matrix (total emissions and
resource use per dollar from domestic activity)...
## 2022-01-31 15:18:57 INFO::Calculating N matrix (total environmental
impacts per dollar)...
## 2022-01-31 15:18:57 INFO::Calculating N_d matrix (total environmental
impacts per dollar from domestic activity)...
## 2022-01-31 15:18:57 INFO::Calculating Rho matrix (price year ratio)...
## 2022-01-31 15:18:57 INFO::Calculating Phi matrix (producer over purchaser
price ratio)...
## 2022-01-31 15:18:57 INFO::Model build complete.
```

Once the model is successfully built, its unique model identifier can be generated with the `generateModelIdentifier` function.

```
generateModelIdentifier(model)
```

```
## [1] "f613d72ded3a900b8476a5945d47359704f6f2d334de82e1f0c211a86b3a97e5"
```

S4 Present a portion of the model crosswalk table

A portion of the `crosswalk` table is presented below to provide a glimpse of the complete `model$crosswalk`.

Table S1: A portion of the model `crosswalk` table. Codes of Agriculture, forestry, fishery, and hunting sectors are presented.

NAICS	BEA_Sector	BEA_Summary	BEA_Detail	USEEIO
11	11	111CA	1111A0	111CA
11	11	111CA	1111B0	111CA
11	11	111CA	111200	111CA
11	11	111CA	111300	111CA
11	11	111CA	111400	111CA
11	11	111CA	111900	111CA
11	11	111CA	112120	111CA
11	11	111CA	1121A0	111CA
11	11	111CA	112300	111CA
11	11	111CA	112A00	111CA
11	11	113FF	113000	113FF
11	11	113FF	114000	113FF
11	11	113FF	115000	113FF
111	11	111CA	1111A0	111CA
111	11	111CA	1111B0	111CA
111	11	111CA	111200	111CA
111	11	111CA	111300	111CA
111	11	111CA	111400	111CA
111	11	111CA	111900	111CA
1111	11	111CA	1111A0	111CA
1111	11	111CA	1111B0	111CA
11111	11	111CA	1111A0	111CA
111110	11	111CA	1111A0	111CA
11112	11	111CA	1111A0	111CA
111120	11	111CA	1111A0	111CA
11113	11	111CA	1111B0	111CA
111130	11	111CA	1111B0	111CA
11114	11	111CA	1111B0	111CA
111140	11	111CA	1111B0	111CA
11115	11	111CA	1111B0	111CA
111150	11	111CA	1111B0	111CA
11116	11	111CA	1111B0	111CA
111160	11	111CA	1111B0	111CA

11119	11	111CA	1111B0	111CA
111191	11	111CA	1111B0	111CA
111199	11	111CA	1111B0	111CA
1112	11	111CA	111200	111CA
11121	11	111CA	111200	111CA
111211	11	111CA	111200	111CA
111219	11	111CA	111200	111CA
1113	11	111CA	111300	111CA
11131	11	111CA	111300	111CA
111310	11	111CA	111300	111CA
11132	11	111CA	111300	111CA
111320	11	111CA	111300	111CA
11133	11	111CA	111300	111CA
111331	11	111CA	111300	111CA
111332	11	111CA	111300	111CA
111333	11	111CA	111300	111CA
111334	11	111CA	111300	111CA
111335	11	111CA	111300	111CA
111336	11	111CA	111300	111CA
111339	11	111CA	111300	111CA
1114	11	111CA	111400	111CA
11141	11	111CA	111400	111CA
111411	11	111CA	111400	111CA
111419	11	111CA	111400	111CA
11142	11	111CA	111400	111CA
111421	11	111CA	111400	111CA
111422	11	111CA	111400	111CA
1119	11	111CA	111900	111CA
11191	11	111CA	111900	111CA
111910	11	111CA	111900	111CA
11192	11	111CA	111900	111CA
111920	11	111CA	111900	111CA
11193	11	111CA	111900	111CA
111930	11	111CA	111900	111CA
11194	11	111CA	111900	111CA
111940	11	111CA	111900	111CA

11199	11	111CA	111900	111CA
111991	11	111CA	111900	111CA
111992	11	111CA	111900	111CA
111998	11	111CA	111900	111CA
112	11	111CA	112120	111CA
112	11	111CA	1121A0	111CA
112	11	111CA	112300	111CA
112	11	111CA	112A00	111CA
1121	11	111CA	112120	111CA
1121	11	111CA	1121A0	111CA
11211	11	111CA	1121A0	111CA
112111	11	111CA	1121A0	111CA
112112	11	111CA	1121A0	111CA
11212	11	111CA	112120	111CA
112120	11	111CA	112120	111CA
11213	11	111CA	1121A0	111CA
112130	11	111CA	1121A0	111CA
1122	11	111CA	112A00	111CA
11221	11	111CA	112A00	111CA
112210	11	111CA	112A00	111CA
1123	11	111CA	112300	111CA
11231	11	111CA	112300	111CA
112310	11	111CA	112300	111CA
11232	11	111CA	112300	111CA
112320	11	111CA	112300	111CA
11233	11	111CA	112300	111CA
112330	11	111CA	112300	111CA
11234	11	111CA	112300	111CA
112340	11	111CA	112300	111CA
11239	11	111CA	112300	111CA
112390	11	111CA	112300	111CA
1124	11	111CA	112A00	111CA
11241	11	111CA	112A00	111CA
112410	11	111CA	112A00	111CA
11242	11	111CA	112A00	111CA
112420	11	111CA	112A00	111CA

1125	11	111CA	112A00	111CA
11251	11	111CA	112A00	111CA
112511	11	111CA	112A00	111CA
112512	11	111CA	112A00	111CA
112519	11	111CA	112A00	111CA
1129	11	111CA	112A00	111CA
11291	11	111CA	112A00	111CA
112910	11	111CA	112A00	111CA
11292	11	111CA	112A00	111CA
112920	11	111CA	112A00	111CA
11293	11	111CA	112A00	111CA
112930	11	111CA	112A00	111CA
11299	11	111CA	112A00	111CA
112990	11	111CA	112A00	111CA
113	11	113FF	113000	113FF
1131	11	113FF	113000	113FF
11311	11	113FF	113000	113FF
113110	11	113FF	113000	113FF
1132	11	113FF	113000	113FF
11321	11	113FF	113000	113FF
113210	11	113FF	113000	113FF
1133	11	113FF	113000	113FF
11331	11	113FF	113000	113FF
113310	11	113FF	113000	113FF
114	11	113FF	114000	113FF
1141	11	113FF	114000	113FF
11411	11	113FF	114000	113FF
114111	11	113FF	114000	113FF
114112	11	113FF	114000	113FF
114119	11	113FF	114000	113FF
1142	11	113FF	114000	113FF
11421	11	113FF	114000	113FF
114210	11	113FF	114000	113FF
115	11	113FF	115000	113FF
1151	11	113FF	115000	113FF
11511	11	113FF	115000	113FF

115111	11	113FF	115000	113FF
115112	11	113FF	115000	113FF
115113	11	113FF	115000	113FF
115114	11	113FF	115000	113FF
115115	11	113FF	115000	113FF
115116	11	113FF	115000	113FF
1152	11	113FF	115000	113FF
11521	11	113FF	115000	113FF
115210	11	113FF	115000	113FF
1153	11	113FF	115000	113FF
11531	11	113FF	115000	113FF
115310	11	113FF	115000	113FF
